# Comprehensive Analysis of the CDPK-SnRK Superfamily Genes in Chinese Cabbage and Its Evolutionary Implications in Plants

**DOI:** 10.3389/fpls.2017.00162

**Published:** 2017-02-10

**Authors:** Peng Wu, Wenli Wang, Weike Duan, Ying Li, Xilin Hou

**Affiliations:** ^1^State Key Laboratory of Crop Genetics and Germplasm Enhancement, Key Laboratory of Biology and Germplasm Enhancement of Horticultural Crops in East China, Ministry of Agriculture, Nanjing Agricultural UniversityNanjing, China; ^2^School of Life Science and Food Engineering, Huaiyin Institute of TechnologyHuaian, China

**Keywords:** CDPK-SnRK genes, *Brassica rapa*, evolutionary conservation, synteny analysis, evolutionary pattern, expression pattern

## Abstract

The CDPK-SnRK (calcium-dependent protein kinase/Snf1-related protein kinase) gene superfamily plays important roles in signaling pathways for disease resistance and various stress responses, as indicated by emerging evidence. In this study, we constructed comparative analyses of gene structure, retention, expansion, whole-genome duplication (WGD) and expression patterns of CDPK-SnRK genes in *Brassica rapa* and their evolution in plants. A total of 49 *BrCPKs*, 14 *BrCRKs*, 3 *BrPPCKs*, 5 *BrPEPRKs*, and 56 *BrSnRK*s were identified in *B. rapa*. All BrCDPK-SnRK proteins had highly conserved kinase domains. By statistical analysis of the number of CDPK-SnRK genes in each species, we found that the expansion of the CDPK-SnRK gene family started from angiosperms. Segmental duplication played a predominant role in CDPK-SnRK gene expansion. The analysis showed that PEPRK was more preferentially retained than other subfamilies and that CPK was retained similarly to SnRK. Among the CPKs and SnRKs, CPKIII and SnRK1 genes were more preferentially retained than other groups. CRK was closest to CPK, which may share a common evolutionary origin. In addition, we identified 196 CPK genes and 252 SnRK genes in 6 species, and their different expansion and evolution types were discovered. Furthermore, the expression of *BrCDPK-SnRK* genes is dynamic in different tissues as well as in response to abiotic stresses, demonstrating their important roles in development in *B. rapa*. In summary, this study provides genome-wide insight into the evolutionary history and mechanisms of CDPK-SnRK genes following whole-genome triplication in *B. rapa*.

## Introduction

Plants are remarkably responsive to a variety of environmental stimuli, including pathogen attack, wounding, cold, drought reception, and fluctuations in incident light (Kudla et al., 2010). Meanwhile, a variety of internal substances also affect plants growth. These external and internal signals compose a complex regulatory network that allows plants to develop in balance. Following the detection of a stress stimulus, various signal transduction pathways are switched on, resulting in physiological changes in the plant cell. As second messengers, calcium ions play an essential role in many important cellular processes, especially under stress conditions (Trewavas and Malhó, 1998; Sanders et al., 1999; Berridge et al., 2000). In plants, transient changes in calcium content in the cytosol (calcium signatures) have been observed during growth, development and stress conditions (Evans et al., 2001; Harper, 2001; Knight and Knight, 2001; Sanders et al., 2002). Intracellular Ca^2+^ signals are produced in plant cells by a variety of stimuli, such as changes in environmental conditions, interaction with microbes, and developmental programs (Bush, 1995; Ehrhardt et al., 1996; Hammond-Kosack and Jones, 1996; Knight et al., 1996; Taylor and Hepler, 1997; Pei et al., 2000; Assmann and Wang, 2001; Murata et al., 2001; McAinsh et al., 2002; Plieth and Trewavas, 2002; Ritchie et al., 2002). Plants have multiple calcium stores, including the apoplast, vacuole, nuclear envelope, endoplasmic reticulum (ER), mitochondria and chloroplasts. Therefore, each stimulus can elicit a characteristic Ca^2+^wave by specifically altering the activity of various differentially localized Ca^2+^ channels, H/Ca^2+^ antiporters, and Ca^2+^ and H^+^ ATPases (Thuleau et al., 1998; Allen et al., 2000; Harmon et al., 2000; Hwang et al., 2000). Different calcium sensors recognize specific calcium signatures and transduce them into downstream effects, including altered protein phosphorylation and gene expression patterns (Sanders et al., 1999; Rudd and Franklin-Tong, 2001).

In eukaryotes, calcium-dependent protein kinases (CDPKs) and most sucrose non-fermenting-1-related kinases (SnRKs) are involved in regulating and decoding Ca^2+^ signals (Assmann and Wang, 2001; Evans et al., 2001; Harmon et al., 2001; Cheng et al., 2002; Fasano et al., 2002; Hrabak et al., 2003; Cho et al., 2009; Kulik et al., 2011). The protein kinases also involved in stress signal transduction in plants are common to all eukaryotic organisms and include mitogen-activated protein kinases (MAPKs), glycogen synthase kinase 3 (GSK3), and S6 kinase (S6K). The CDPK-SnRK superfamily consists of seven types of protein kinases, which differ in the regulatory domains they contain (Harmon et al., 2001). CDPKs (also named CPKs) are activated by the binding of calcium to their calmodulin-like regulatory domains. The carboxyl terminal domains of CRKs (CDPK-related kinases) have sequence similarity to the regulatory domains of CPKs but do not bind calcium. PEPRKs (PEP carboxylase kinases) contain only one catalytic domain (Harmon et al., 2001). PPCKs (PEPC kinases) have a carboxyl-terminal domain that has no similarity to that of any other member of the superfamily (Hrabak et al., 2003). CCaMKs (calcium- and calmodulin-dependent protein kinases) bind both calcium ions and the calcium/calmodulin complex, whereas CaMKs (calmodulin-dependent protein kinases) bind the calcium/calmodulin complex but not calcium (Hrabak et al., 2003). In addition, there are the classical SNF1-type kinases from yeast; Halford and Hardie (1998) proposed the name SNF1-related kinase (SnRK) for this group and recognized three subgroups: SnRK1, SnRK2, and SnRK3 (Harmon et al., 2001). However, CaMK and CCaMK are absent from *Arabidopsis* (Hrabak et al., 2003). All members of the CDPK-SnRK gene superfamily have kinase domains of similar length and sequence and a similar general organization, with the kinase domains at or near the N-terminus, then the junction domains, followed by the regulatory domains (Harmon, 2003; Hrabak et al., 2003).

The plant CPKs characterized to date play substantive roles in diverse physiological processes. These processes include tolerance to salt, cold, and drought stress in rice (Saijo et al., 2000), the defense response in tobacco (Romeis et al., 2000), the accumulation of storage starch and protein in immature seeds of rice (Asano et al., 2002), the regulation and development of nodule number in *Medicago truncatula* (Gargantini et al., 2006), and the response to ABA in *Arabidopsis* (Choi et al., 2005) (38). The original, systematic report on the CPK genes family in *Arabidopsis thaliana* identified 34 CPK genes family members (Choi et al., 2005) and was followed by research in rice (*Oryza sativa*) (Ray et al., 2007) and wheat (*Triticum aestivum*) (Li et al., 2008). Recently, genome-wide analyses of the CPK gene family have been reported in maize (*Zea mays*) (Kong et al., 2013) and poplar (*Populus trichocarpa*) (Zuo et al., 2013). Meanwhile, more and more investigations of CPK genes have also involved horticultural plants, such as alfalfa (Davletova et al., 2001), potato (Raíces et al., 2003), strawberry (Llop-Tous et al., 2002), and tomato (Chico et al., 2002). Furthermore, research using transgenic plants has revealed the biological functions of a few CPK genes in higher plants. Transgenic rice constitutively overexpressing *OsCPK7* or *OsCPK13* showed enhanced tolerance to cold, salt, and drought stress (Saijo et al., 2000; Komatsu et al., 2007). In tobacco, CPK-silenced plants displayed a reduced and delayed hypersensitive response to the fungal Avr9 elicitor (Romeis et al., 2001). *GhCPK1* was the first cotton CPK gene to be identified and was considered to play a role in the calcium signaling events associated with fiber elongation (Huang et al., 2008). *Arabidopsis thaliana CPK23* (*AtCPK23*) is a positive regulator of the response to drought and salt stress (Ma and Wu, 2007), whereas *AtCPK6* may be crucial in positively regulating methyl-jasmonate signaling in guard cells (Munemasa et al., 2011). In addition, the overexpression of rice (*Oryza sativa*) *CPK7* (*OsCPK7*) significantly improves resistance to cold (Komatsu et al., 2007). Phytohormones are involved in the responses to abiotic stresses; therefore, the expression levels of members of the CPK gene family has also been shown to be regulated after treatment with various phytohormones, such as ABA, auxin and jasmonic acid. Recently, *Zea mays CPK11* was reported as a component of the jasmonic acid signaling pathway, and its concentration in cells was observed to increase in response to wounding and touch (Szczegielniak et al., 2012).

Sucrose non-fermenting-1-related protein kinase (SnRK) is homologous to SNF1 and AMP-activated protein kinase (AMPK), which is widely distributed in plants and is involved in a variety of signaling pathways. SnRK is the key switch in plant sugar signaling, stress, seed germination and seedling growth. SNF1 of yeast, AMPK of mammals and SnRK1 of plants are homologous, belonging to the SNF1 protein kinase superfamily. SNF1 was found in yeast (*Saccharomyces cerevisiae*) originally (Alderson et al., 1991). In yeast, glucose regulates the protein-protein interaction, substrate specificity and subcellular localization of the SNF1 subunit that modulates SNF1 kinase activity, resulting in the phosphorylation of activators and repressors that control transcription of multiple genes in metabolic pathways required for the utilization of alternative energy sources. In the eukaryote, SNF1 protein kinase is very strongly conserved. Many SNF1 analogs have been identified in plants. SnRK1 was discovered initially in rye (*Secale cereale* L.) (Alderson et al., 1991). At present, some members of the SnRK1 subfamily have been found in variety of model plants and some important crops, such as *Arabidopsis thaliana*, rye, barley (*Hordeum vulgare*), potatoes (*Solanum tuberosum*), tobacco, beets, etc. It may exist in all plants (Halford and Hardie, 1998; Halford et al., 2003). Studies have shown that SnRK1 is the key switch in plant sugar signaling. In addition, the regulation of glucose metabolism, hormonal regulation and sugar signaling is directly related to signal transduction (Kleinow et al., 2000; Jossier et al., 2009; Mathieu et al., 2009). SnRK2s are a plant-specific Ser/Thr protein kinase family. All of the members have a conserved N-terminal catalytic domain similar to that of SNF1/AMPK-type kinases and a short C-terminal regulatory domain that is not highly conserved. Prior to 2000, there were only a small number of studies indicating that ABA and abiotic stresses induced the expression of some *SnRK2* genes (Anderberg and Walker-Simmons, 1992; Holappa and Walker-Simmons, 1995). In 2000, SnRK2s began to be recognized as enzymes involved in abiotic stress signal transduction in plants (Li et al., 2000). By 2003, 10 SnRK2 genes had been identified and were renamed *SnRK2.1* through *SnRK2.10* (Hrabak et al., 2003). In 2009, independently, two laboratories obtained a triple *SnRK2.2/2.3/2.6* mutant. *SnRK2.2/2.3/2.6* triple-mutant plants are nearly completely insensitive to ABA, which was used to establish the role of ABA-dependent SnRK2s in the plant response to water deficit, seed maturation, and germination. These reports indicate that *SnRK2.2/2.3/2.6* function as primary positive regulators and suggest that ABA signaling is controlled by the dual modulation of *SnRK2.2/3/6* and group A PP2Cs (Fujii and Zhu, 2009; Fujii et al., 2009; Nakashima et al., 2009). SnRK3 is a protein kinase in plants, called calcineurin B-like calcium sensor-interacting protein kinase (CIPK) (Kim et al., 2000). CIPK interacts with the calcium-binding protein SOS3, SCaBPS and CBL (calcineurin B-like calcium sensor). Studies have shown that CIPK and an upstream complex of CSL interactions are involved in salt stress, sucrose and ABA signal transduction (Imamura et al., 2008). In *Arabidopsis*, PKS3, PKS18 and CIPK3 of the SnRK3 family can regulate plant growth, stomatal opening and closing and seed germination under ABA treatment (Kim et al., 2003). Arabidopsis AtCIPK1 forms complexes with AtCBL1 and AtCBL9, regulating ABA-independent and ABA-dependent pathways, respectively (D'Angelo et al., 2006). AtCIPK3 regulates ABA and cold signal transduction pathways (Kim et al., 2003). Girdhar's study showed that CBL9 interacted with CIPK3 to regulate the ABA pathway, and this finding was validated in a yeast two-hybrid experiment (Pandey et al., 2008).

During their evolution, plants have substantially altered their phenotypes to adapt to environmental changes by transforming the form and function of genes. Gene duplication, even a whole-genome duplication (WGD), offers the chance for genes to change (Rensing, 2014). Angiosperm genome evolution is characterized by polyploidization through WGD followed by diploidization, which is typically accompanied by considerable homoeologous gene loss (Stebbins, 1950). After duplication, one copy of the gene might either becomes non-functional (pseudogenized or silenced, also called gene death) or acquire a novel function (neofunctionalization). Alternatively, the two duplicates might divide the original function of the gene (Innan and Kondrashov, 2010). Preliminary analyses revealed that gene duplication and subsequent divergence are the main contributors to evolutionary momentum (Ohno et al., 1968; Chothia et al., 2003). The genome of *A. thaliana* has experienced a paleohexaploidy (β) duplication shared with most dicots and two subsequent genome duplications (α and γ) since its divergence from *Carica papaya*, along with rapid DNA sequence divergence and extensive gene loss (Bowers et al., 2003). In *A. thaliana*, some duplicated regions found in CDPK-SnRK protein kinases indicated that CDPK-SnRK protein kinases are paralogs that arose by divergence after genome duplication events (Hrabak et al., 2003). The CPK genes of *Arabidopsis* and maize have undergone both segmental and tandem duplication, contributing to the expansion of the CPK family. In *Populus*, however, segmental duplication played a predominant role in the expansion of CPK genes (Zuo et al., 2013). In addition, tandem duplication of CPK genes has not occurred in the rice genome (Asano et al., 2005).

In this study, we constructed a comprehensive comparative analysis of CDPK-SnRK genes, including phylogenetic relationships, gene structures, chromosome distribution, gene retentions, gene expansions, gene duplication and gene expression patterns, in different tissues to characterize the divergences in composition, expansion, and expression. First, we identified 555 *CPKs*, 120 *CRKs*, 5 *PPCKs*, 14 *PEPRKs*, and 697 *SnRK*s in 16 plant species. Second, we conducted a comparative genomic analysis of these genes with 16 other plant and species found that the expansion of the CDPK-SnRK family from angiosperms mainly relied on WGDs. Third, PEPRK genes were more preferentially retained than other subfamilies and CPK genes were retained similarly to SnRK genes during diploidization following WGT in *B. rapa*. Fourth, during the course of evolution, CPK appeared most recently and expanded most rapidly. Fifth, the expressions of CDPK-SnRK genes are dynamic in different tissues as well as in response to abiotic stresses, demonstrating their important roles in development in *B. rapa*. This study is the first report on CDPK-SnRK genes in *B. rapa*. and extends our understanding of the roles of the CDPK-SnRK gene superfamily in evolution and stress responses.

## Results

### Identification and classification of the CDPK-SnRK superfamily of protein kinases in *Brassica rapa* and comparative analyses

In this study, genome-wide analysis of CDPK-SnRK gene family has been performed on the basis of the completed *B. rapa* genome sequence (Wang et al., 2011). Based on previously reported methods (Harmon et al., 2001; Hrabak et al., 2003), the homogeneous candidate CDPK-SnRK genes between *Brassica rapa* and other species were identified by BLASTP (Supplementary Table 1). Subsequently, all candidate protein sequences were subjected to Pfam and SMART analyses. Finally, we identified 49 *BrCPKs*, 14 *BrCRKs*, 3 *BrPPCKs*, 5 *BrPEPRKs*, and 56 *BrSnRK*s named according to nomenclature proposed for CDPK-SnRK genes (Supplementary Table 2).

**Figure 1:**
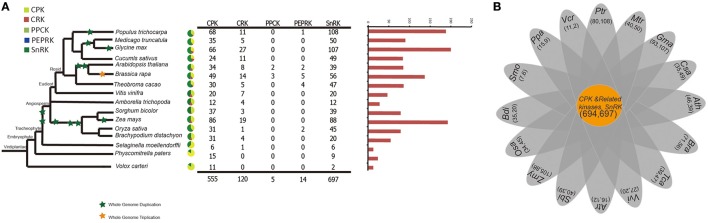
**A comparative analysis of CDPK-SnRK genes in plants. (A)** The evolutionary relationships and the numerical details of the CDPK-SnRK superfamily of each species. **(B)** The Venn diagram shows the number of common gene families and genes in 16 plants.

To better understand the expansion and evolutionary history of CDPK-SnRK genes in *B. rapa*, genes were also identified in 16 other species representing the major clades of plants. The evolutionary relationships of the species and the number of CDPK-SnRK genes are shown in Figure 1A. The data show that *Glycine max* contained the highest number of CDPK-SnRK genes (200), followed by *Z. mays* (193) and *M. truncatula* (188) (Figure 1A). However, *A. trichopoda*, a basal angiosperm species that was the single living representative of the sister lineage to all other extant flowering plants, contained the lowest number of CDPK-SnRK genes (28) in *Angiospermae*. The reason is that it originated prior to the split of eudicots and monocots and has not experienced any whole genome duplication (WGD), while the other 12 angiosperms had several rounds of WGDs/triplications after their split from *A. trichopoda*. Furthermore, the number of CDPK-SnRK genes in algae, Bryophyta and Pteridophyta was less than that in *Angiospermae*. This phenomenon was also caused by several WGD events that occurred during angiosperm evolution (Figure 1B). These results indicated that the expansion of the CDPK-SnRK family from angiosperms mainly relied on large-scale DNA rearrangements, namely, WGDs. The elevated duplication frequency and increased retention of CDPK-SnRK genes also contributed to neofunctionalization and caused them to gain important functions in angiosperm development.

### Characteristics of structure, and expansion analysis of BrCDPK-SnRK proteins

**Figure 2:**
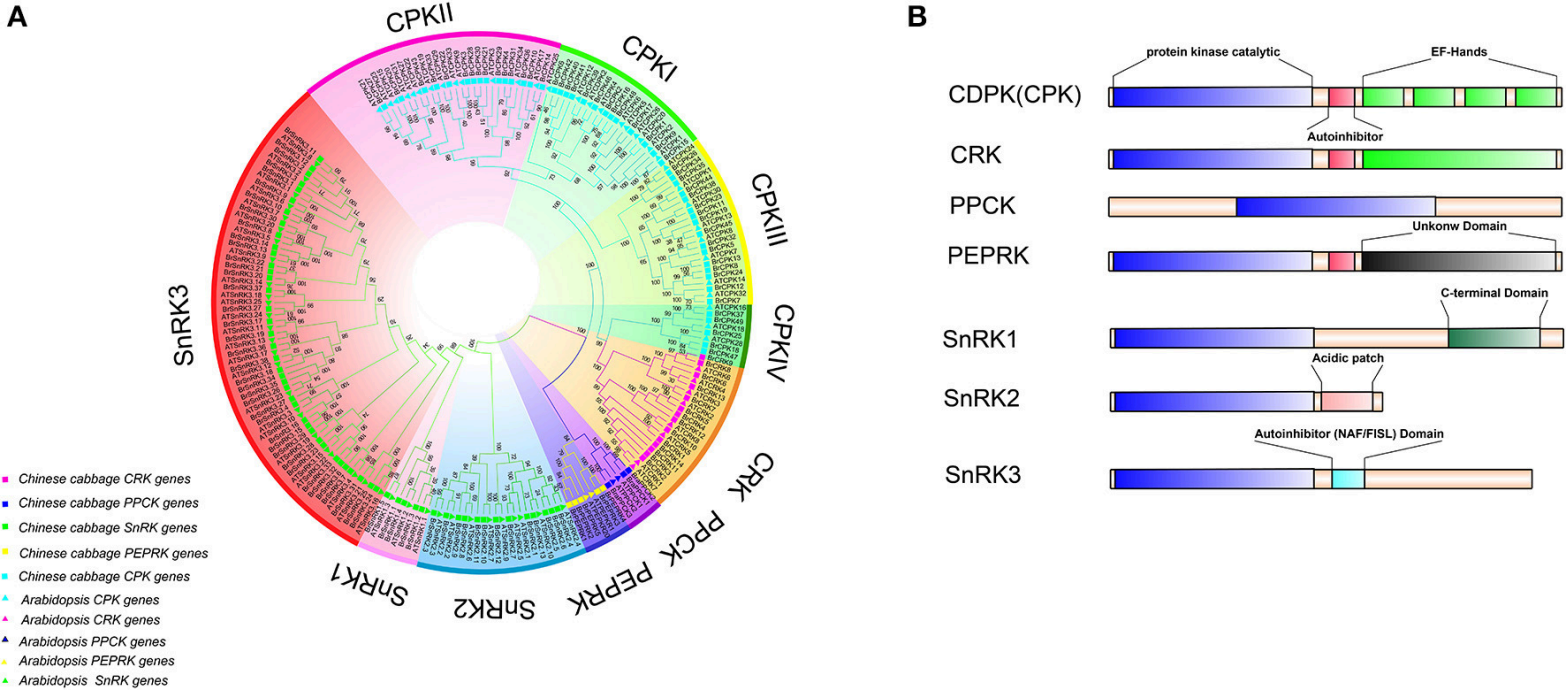
**Phylogenetic tree and major domains of CDPK-SnRK proteins**. **(A)** Phylogenetic analysis of 212 CDPK-SnRK proteins from Chinese cabbage (127) and *Arabidopsis* (85) showing similar groups in all of the plant species. In total, 10 clades with different colors that were formed by CDPK-SnRK proteins are also marked. **(B)** The kinases are drawn with the N-terminus to the left and C-terminus to the right. Catalytic domains (blue boxes) have been aligned to facilitate comparison. Auto-inhibitory domains are shown as red boxes. EF-hands are shown as green boxes within the regulatory domains. The SnRK1 C-terminus domains are shown as dark green boxes. Acidic patch domains are shown as pink boxes. The SnRK3 NAF/FISL domains are shown as dark and light blue boxes.

To investigate the extent of lineage-specific expansion of the CDPK-SnRK genes in *B. rapa*, phylogenetic trees were constructed using the maximum likelihood method (Figure 2A). The phylogenetic tree showed that all the CDPK-SnRK genes were clustered into five distinct gene classes (CPK, CRK, PEPRK, PPCK, SnRK) (Figure 2A), while the CPK family was divided into four groups (I, II, III, and IV) and the SnRK family was classified into three groups (SnRK1, SnRK2, and SnRK3), consistent with the reports in *A. thaliana* (Hrabak et al., 2003). In *B. rapa*, the CPK, CRK, PEPRK, PPCK, and SnRK gene families contained 49 members, 14 members, 3 members, 5 members, and 56 members, respectively, whereas in *A. thaliana*, the CPK, CRK, PEPRK, PPCK, and SnRK families contained 34 members, 8 members, 2 members, 2 members, and 39 members, respectively. Next, the synteny of CDPK-SnRK genes between *A. thaliana* and three subgenomes in *B. rapa* was analyzed. There were 34 *CPK*, 8 *CRK*, 2 *PEPRK*, 2 *PPCK*, and 38 *SnRK* genes on the conserved collinear block (Supplementary Table 5). Meanwhile, 2 *CPKs*, 2 *CRKs*, 1 *PEPRK*, 1 *PPCK*, and 3 *SnRK*s were retained completely; conversely, 4 *CPKs* and 3 *SnRKs* from *B. rapa* were lost. Due to the *Brassica*-specific WGT event, the gene numbers of these classes in *B. rapa* were greater than those in *A. thaliana*.

**Figure 3:**
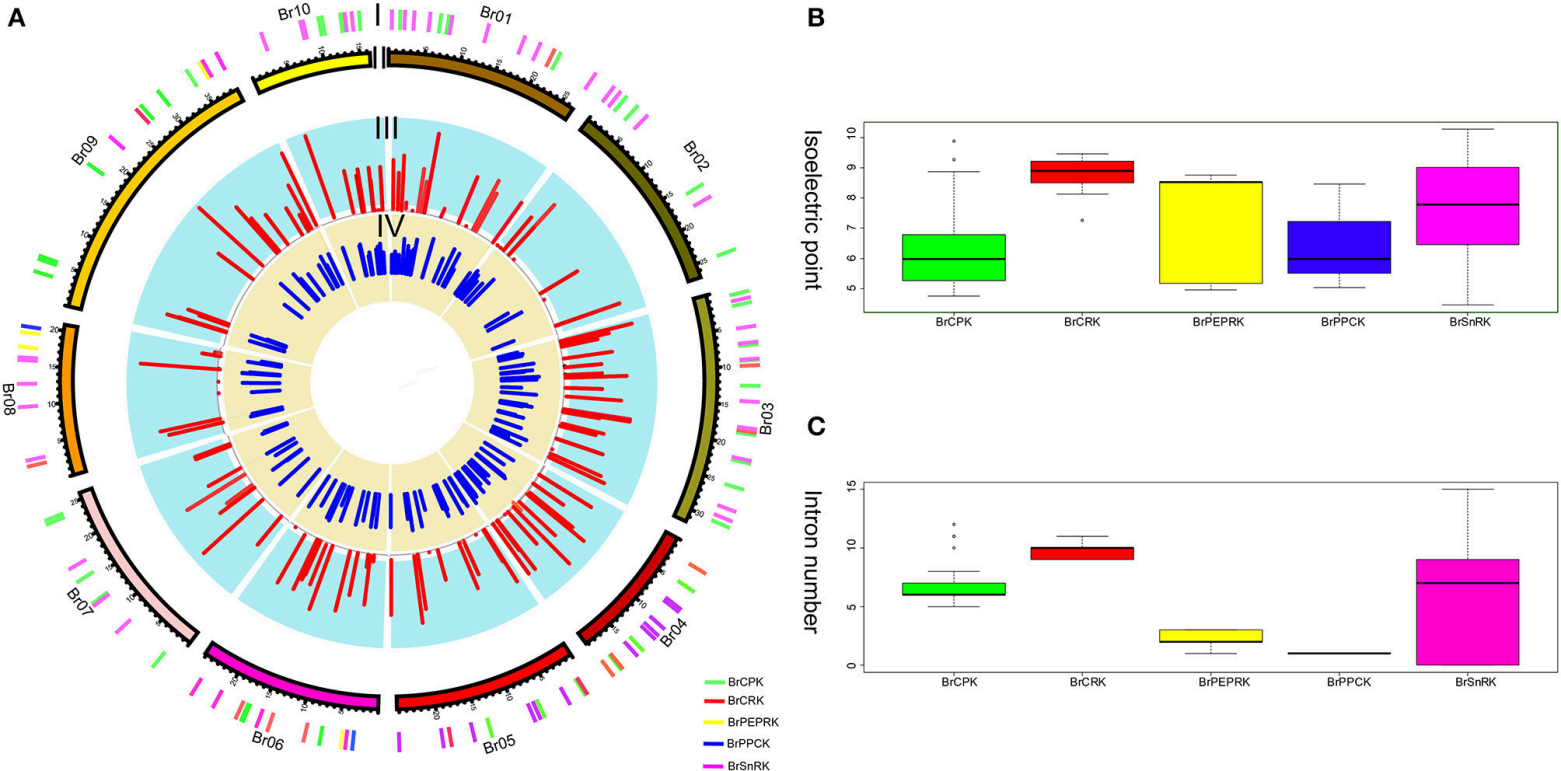
**Characteristics of isoelectric points and introns among BrCDPK-SnRK**. **(A)** I indicates CDPK-SnRK genes within the *B. rapa* Genome. II indicates the *B. rapa* chromosome karyotype. III represents the number of introns (red) of CDPK-SnRK genes. IV indicates the Pi value (blue) of BrCDPK-SnRK genes. **(B)** The Pi value among gene classes BrCPK, BrCRK, BrPEPRK, BrPPCK, and BrSnRK. **(C)** The number of introns among gene classes BrCPK, BrCRK, BrPEPRK, BrPPCK, and BrSnRK.

Furthermore, the different domain architectures, motif compositions and gene structures of CDPK-SnRK were analyzed (Figure 2B, Figure S1). All members of the CDPK-SnRK superfamily have a kinase domain of similar length and sequence, with the kinase domains at or near the N-terminus, then the junction domains, followed by the regulatory domains (Figure 2B). Although CPK proteins have a functional kinase domain coupled with regulatory calcium-binding EF-hands, the C-terminal domains (EF-hands) of CRK proteins contain apparently degenerate calcium-binding sites with no function. Meanwhile, 10 conserved motifs were detected in BrCPK, BrCRK, BrPEPRK, BrPPCK, and BrSnRK, respectively (Figure S3). All BrCDPK-SnRK proteins had highly conserved kinase domains, which corresponded to motifs 1-4,7,9 in BrCDPK and motifs 1-4,6,7,9 in BrSnRK, whereas motif 8 was found in *BrSnRK3.1*, corresponding to the NAF/FISL domains (Figures S1A,B). The amino acid sequence of BrCDPK-SnRK was aligned with AtCDPK-SnRK protein sequences from five gene classes. In CPK, CRK, PEPRK, PPCK, and SnRK, higher sequence similarities were identified in the N-terminus, which corresponded to the conserved kinase domain (Figure S2). In addition, variable gene structures of BrCDPK-SnRK were observed. As shown in Figures 3A,C, the intron numbers of the BrCPK genes ranged from 5 to 9 with a median of 6, while the BrSnRK genes ranged from 0 to 15 with a median of 7. Interestingly, we found that 22 BrSnRK3 genes have no introns. The theoretical pI of the BrCPK gene family ranged from 4 to 9 with a median of 6, but BrSnRK proteins showed a pI range from 2 to 11 and with a median of 8 (Figure 3B). Other classes had complex theoretical pI ranging in value from 4 to 9 (Figure 3B).

### Different retention of CDPK-SnRK genes following WGT in *Brassica rapa*

**Figure 4:**
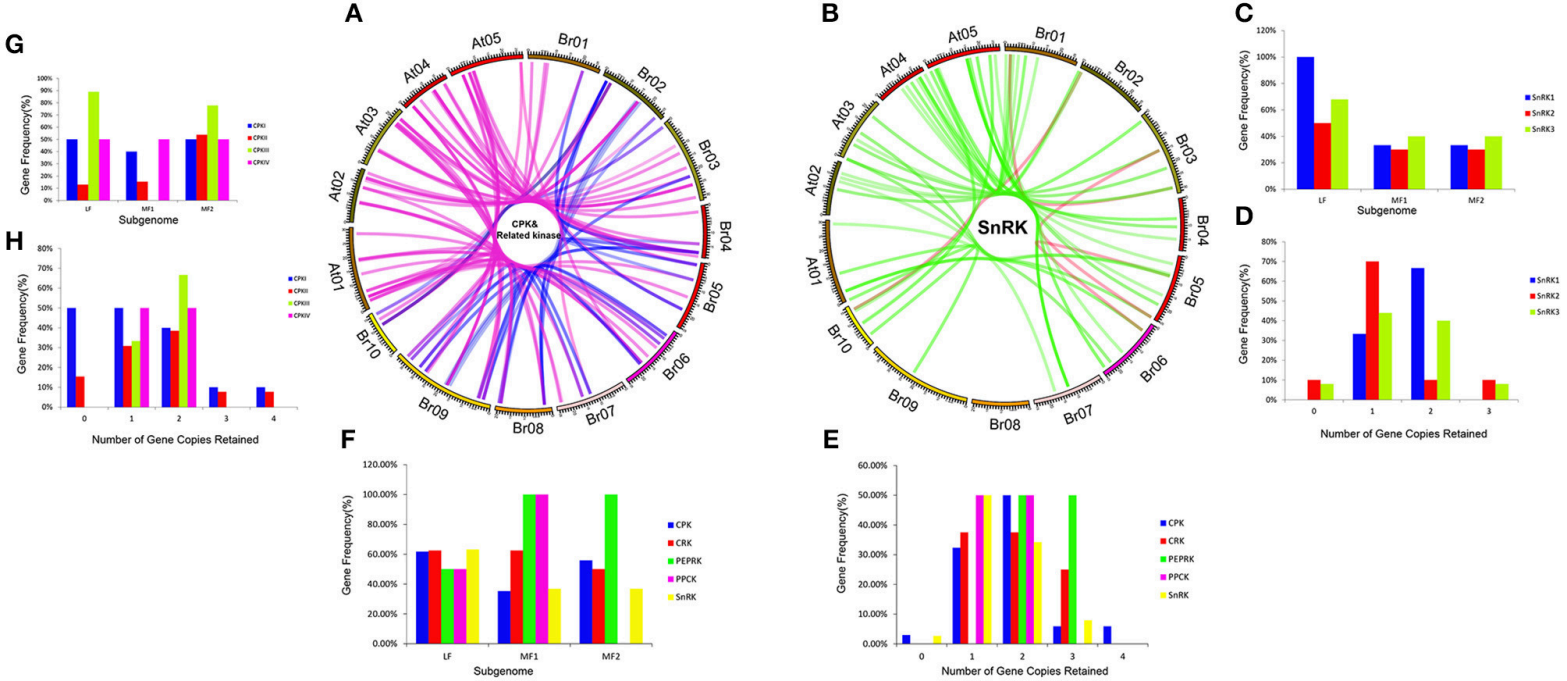
**CDPK-SnRK homologous genes in the segmental syntenic regions of the genomes of ***Brassica rapa*** and ***Arabidopsis thaliana*** and their different retention. (A)** CPK, CRK, PEPRK, and PPCK syntenic gene lines are shown between the 10 *B. rapa* chromosomes (Br01-Br10) and the five *Ar. Thaliana* chromosomes (At01-At05). The pink lines represent the syntenic gene pairs between Chinese cabbage and *Arabidopsis*; the blue lines represent the syntenic genes in Chinese cabbage. **(B)** SnRK syntenic gene lines are shown between the 10 *B. rapa* chromosomes and the five *A. thaliana* chromosomes. The green lines represent the syntenic genes pairs between Chinese cabbage and *Arabidopsis*; the red lines represent the syntenic genes in Chinese cabbage. **(C)** Retention of homoeologs of SnRK genes in the three subgenomes (LF, MF1, and MF2) in *B. rapa*. LF: least fractionized subgenome; MF1: moderately fractionized subgenome; MF2: most fractionized subgenome. **(D)** Copy numbers of SnRK genes after genome triplication and fractionation in *B. rapa*. **(E)** Copy numbers of CDPK-SnRK genes after genome triplication and fractionation in *B. rapa*. **(F)** Retention of homoeologs of CDPK-SnRK genes in the three subgenomes (LF, MF1, and MF2) in *B. rapa*. **(G)** Retention of homoeologs of CPK genes in the three subgenomes (LF, MF1, and MF2) in *B. rapa*. **(H)** Copy numbers of CPK genes after genome triplication and fractionation in *B. rapa*.

To investigate different retention in CPK, CRK, PEPRK, PPCK, and SnRK during *B. rapa* WGT events, 44/49, 14/14, 3/3, 5/5, and 50/56 were located in the syntenic regions, respectively (Figures 4A,B and Supplementary Table 3). The results demonstrated that 43% (44/102) of the CPK genes were retained in the syntenic regions, relative to 44% (50/114) of the SnRK genes. The retention rates of CRK, PEPRK and PPCK are 58% (14/24), 50% (3/6), and 83% (5/6), respectively (Figure 4F). Additionally, we counted gene copies and analyzed the distribution of the three subgenomes by comparing the retention of CPK, CRK, PEPRK, PPCK, and SnRK (Figure 4E). The result showed that all PEPRK genes had more than two copies retained, which is more than the retention of the other subfamilies (42%). However, 3% of the CPK and SnRK genes were completely lost. Next, the proportions of CPK and SnRK genes retained were higher in the least fractionated (LF) subgenome than in the medium fractionated (MF1) and most fractionated (MF2) subgenome, consistent with a previous report showing that the degree of retained genes in these three subgenomes (LF, MF1, and MF2) was decreased (Wang et al., 2011). In contrast, the PEPRK and PPCK families were retained more in the MF1 subgenome than in the LF subgenome (Figure 4F). In summary, the results confirmed that PEPRK genes were more preferentially retained than other subfamilies and that CPK genes were retained similarly to SnRK genes during diploidization following WGT in *B. rapa*.

Furthermore, the retention rates of four CPK (*CPKI, II, III, IV*) and three SnRK (*SnRK1, 2, 3*) groups were observed. As show in Figure 4H, 67% of CPKIIIs and SnRK1s had more than two copies retained, which is greater than the retention of the other groups (Figures 4H,D). In addition, the proportion of CPKIIIs and SnRK1s retained was higher in the LF subgenome than in the other subgenomes, which once again confirmed that CPKIII and SnRK1 genes were more preferentially retained than other groups (Figures 4C,G).

### Chromosome distribution, *Ks* and duplication analysis of the CDPK-SnRK genes in *B. rapa*

**Figure 5:**
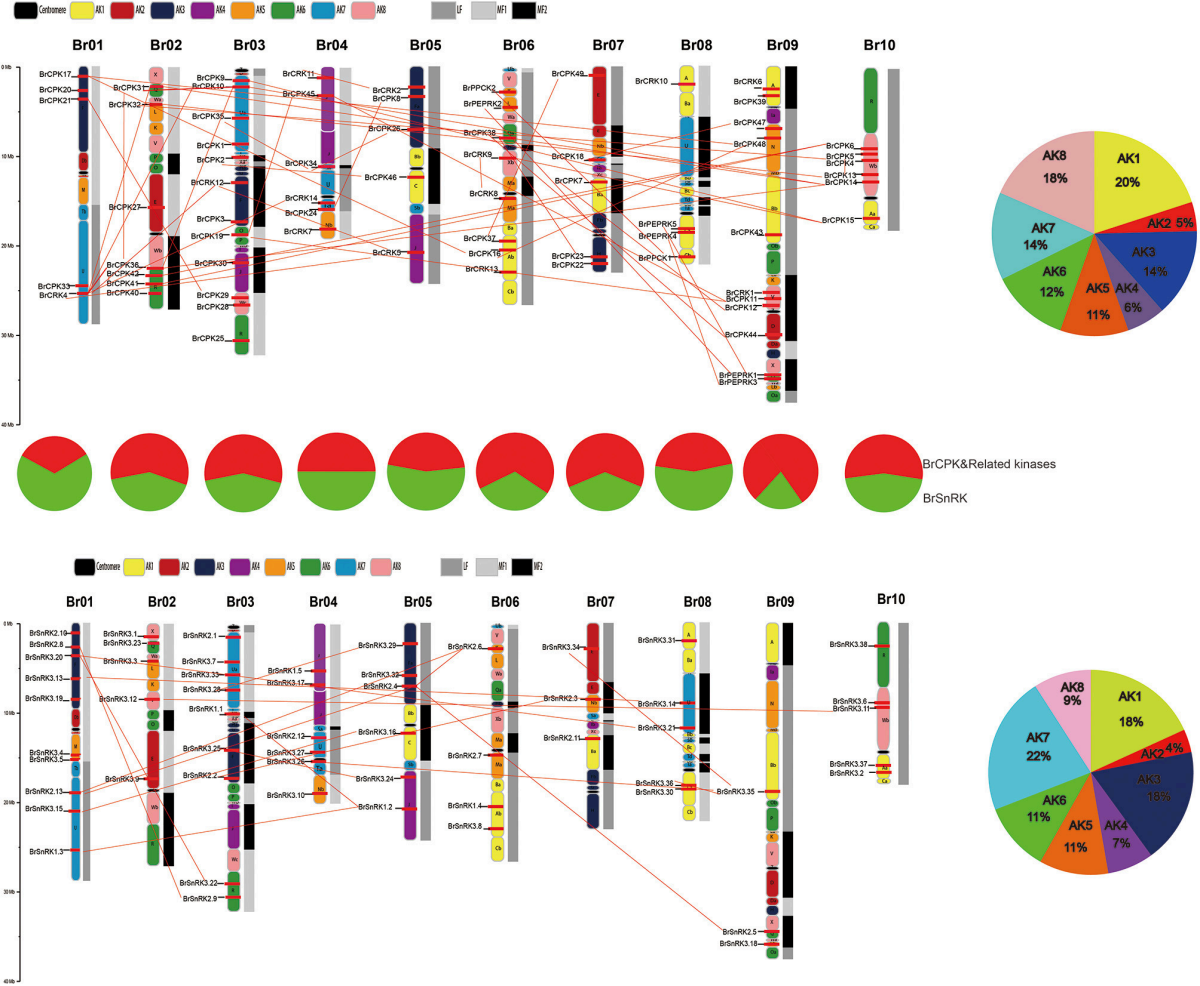
**The features of the CDPK-SnRK genes in ***B. rapa*****. The distribution of BrCDPK-SnRK genes on 10 chromosomes. The 8 ancestral blocks and three subgenomes, including the least fractionated (LF), medium fractionated (MF1), and most fractionated (MF2) subgenomes, were plotted as described by Cheng et al. (2013). AK represents the ancestral karyotype. The red lines connect the duplicated BrCDPK-SnRK genes.

All BrCDPK-SnRK genes could be mapped onto 10 chromosomes of Chinese cabbage with a non-random distribution, except *BrPPCK3*, which is located in Scaffold000191 (Figure 5). On every chromosome, the proportion of BrCPK genes was similar to that of BrSnRK genes. However, Chromosome 09 contained more BrCPK genes (11 genes) than BrSnRK genes, whereas chromosomes 01 had the opposite. *B. rapa* shares two WGDs (WGD: α and β) and one whole-genome triplication event (WGT: γ) in its evolutionary history with *Arabidopsis* but has undergone an additional WGT event. Therefore, the *B. rapa* genome was further divided into three differentially fractionated subgenomes (LF, MF1, MF2), of which LF contained more BrCPK genes and BrSnRK genes than either of the other two subgenomes. In addition, the 24 conserved ancestral genomic blocks (labeled A–X) in the *B. rapa* genome were reconstructed according to previous reports (Cheng et al., 2013). The color coding of these blocks was based on their positions in a proposed ancestral karyotype (AK1-8). We also found that most of the BrCPK genes cluster together in a region of AK1 (20%), whereas BrSnRK genes belonged to AK1 (18%) and AK3 (18%).

**Figure 6:**
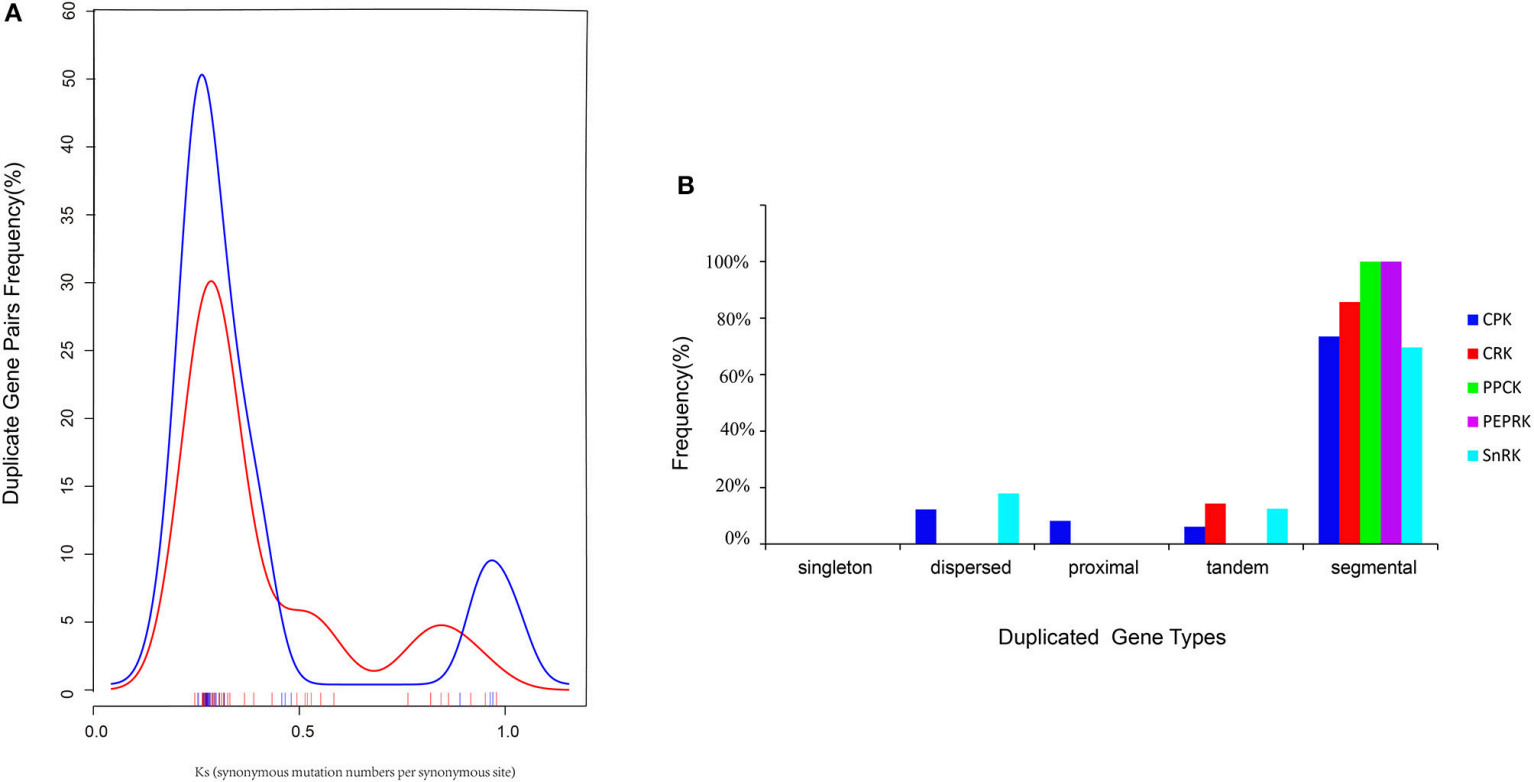
**Pairwise comparison of the ***Ks*** values and types for the duplicated ***CDPK-SnRK*** gene pairs in ***Brassica rapa***. (A)** The distribution of the *Ks* values for CDPK-SnRK genes between *A. thaliana* and *B. rapa*. **(B)** The different duplicated types (singleton, dispersed, proximal, tandem, and segmental) of CDPK-SnRK were counted in *B. rapa*.

Furthermore, the types were identified by the MCScanX program, and the divergence times of the duplicated genes were estimated by calculating the synonymous substitution rates (*Ks*) and non-synonymous substitution rates (*Ka*). In total, 70 *BrCDPK-SnRK* duplicated gene pairs were analyzed (Supplementary Table 4). *BrCPK* (74%), *BrCRK* (86%), *BrPEPRK* (100%), *BrPPCK* (100%), and *BrSnRK* (70%) duplicated gene pairs were segmental duplications (Figure 6B), and all the duplicated *BrCDPK-SnRK* gene pairs had *Ka/Ks* ratios less than 1, indicating the purifying selection of these genes (Figure 6A, Supplementary Table 4). To understand the divergence time, the *Ks* values of the BrCPK genes ranged from 0.3 to 0.5 and had a mean of ~0.34 (~11 *Myr*), while the BrSnRK genes ranged from 0.2 to 0.55 and focused on ~0.25 (~8.5 *Myr*; Figures 2A,B, 6A). The divergence time of BrSnRK duplicated gene pairs was 8.49 MYA, which indicates that their divergence occurred during the *Brassica* triplication events (5 ~ 9 *MYA*). The divergence times obtained for the BrCPK duplicated gene pairs ranged from 10 to 16.6 *MYA*, indicating that these duplications occurred during the divergence of Chinese cabbage and *Arabidopsis* (9.6–16.1 *MYA*).

### Evolution pattern of CDPK-SnRK genes in plants

To investigate the evolution of the CDPK-SnRK family in the plant kingdom, we selected 13 Angiospermae (8 eudicots, 4 monocots and one basal angiosperm), 3 Gymnospermae, 1 Pteridophyta, 1 Bryophyta, and 1 Chlorophyta species for comparative analysis (Figure S3). We constructed a phylogenetic tree of the *CDPK-SnRK* genes to analyze the evolutionary relationships of these species. The phylogenetic tree showed that the CDPK-SnRK genes formed five gene classes (CPK, CRK, PEPRK, PPCK, and SnRK), which is consistent with the result for *B. rapa* and *A. thaliana*. Meanwhile, we found that no CRK, PEPRK, or PPCK genes were detected in *Volvox carteri*. Therefore, the CRK, PEPRK, and PPCK gene families may only exist in land plants. To further determine the relationship among the five groups, the genetic distance was analyzed (Figures S4A,B). The box plot shows the genetic distance of CPK vs. CRK, PEPRK, and PPCK, which was smaller than SnRK vs. these groups (Figure S4B). Notably, the genetic distance between CPK and CRK was smaller than that between CPK and PEPRK or CPK and PPCK. These results indicate that CPK has a closer relationship with CRK, PEPRK, and PPCK, especially the CRK closest to CPK, which is consistent with previous reports that plant CPK and CRK may share a common evolutionary origin (Hrabak et al., 2003).

To further investigated the footprint of the CPK CRK, PPCK, PEPRK, and SnRK families, we selected four angiosperms (*C. papaya, P. trichocarpa, A. trichopoda*, and *V. vinifera*). The reason is that *Vitis vinifera, P. trichocarpa*, and *C. papaya* did not undergo α and β duplications and *A. trichopoda*, a basal angiosperm, did not undergo the γ duplication event (Jiao et al., 2011b; Albert et al., 2013; Lee et al., 2013). Phylogenetic trees in each species were constructed (Figures S5, S6). In each species, the CPK family was divided into four clades (*CPKI, CPKII, CPKIII*, and *CPKIV*), and SnRK was divided into three clades (SnRK1, SnRK2, and SnRK3). CPK, CRK, and SnRK were found to exist in *Amborella trichopoda*, which indicates that these three groups originated from duplication events prior to the γ event. However, PEPRK appeared between the salicoid duplication and the γ event. The PPCK family exists in *P. trichocarpa*, which indicates that it originated from duplication events prior to the salicoid duplication. Furthermore, due to the salicoid duplication and *Brassica*-specific WGT events, there were more CDPK-SnRK gene family members in *P. trichocarpa* and *B. rapa* than in other species (Tuskan et al., 2006; Wang et al., 2011). In general, during the course of evolution, CPK appeared most recently and expanded most rapidly. Above all, we inferred a possible evolutionary footprint of the CPK family (Figure S7).

**Figure 7:**
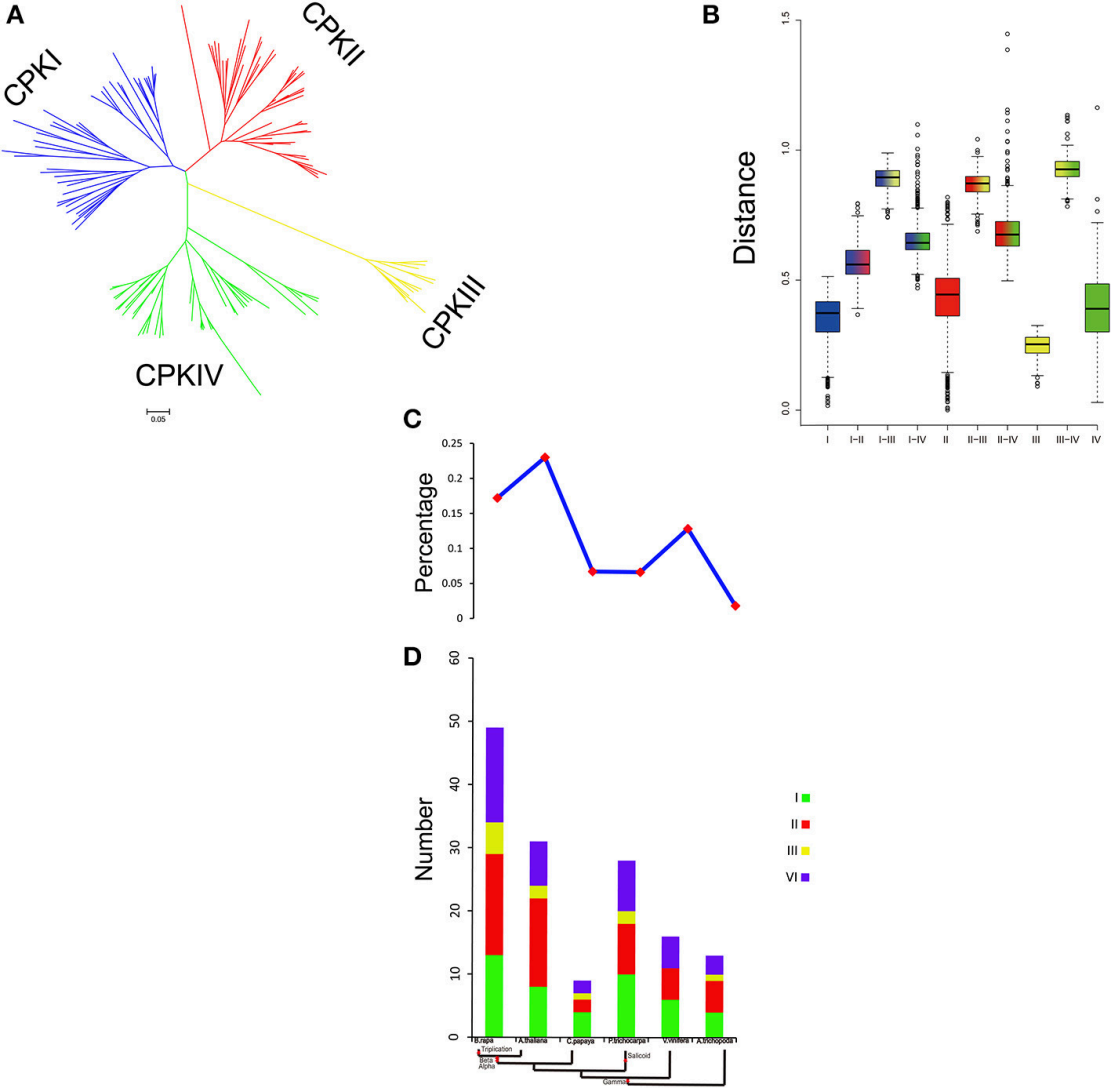
**Analysis of CPK gene evolution. (A)** Phylogenetic relationships among CPK genes; **(B)** genetic distance among the different groups of CPK genes; **(C,D)** comparison of the percentage of CPK genes and copy numbers of CPK genes; CPK genes in representative species.

The family size and the percentage of CPKs in five plants suggested that CPKs expanded rapidly during evolution and further expanded in the Brassicaceae (Figure 7). WGD is known to have important impacts on the expansion and evolution of gene families in plant genomes. However, along with the gradual increase in the CPK percentage, the genes of group III were completely lost in *V. vinifera* (Figure 7C). Compared with other groups, the expansion of group III was more unstable. To further investigate the relationship among the four groups, the genetic distance was analyzed. The results indicated that the genetic distance between CPKI and CPKIII was shorter than that between CPKI and CPKIV or that between CPKI and CPKII, and the genetic distance between CPKII and CPKIV was shorter than that between CPKII and CPKIII (Figure 7B). In summary, we inferred a possible evolutionary footprint of the CPK family. Before the γ event, all groups in the family (CPKI, CPKII, CPKII, CPKIV) had already appeared. The gene family further expanded within Brassicaceae. Thus, the CPK family almost doubled in size in the *B. rapa* genome compared with that of *A. trichopoda* through three duplications, one triplication, and fractionation. The expansion of groups I, II, and IV played a major role in the expansion of the CPK gene family.

### Comparative expression pattern analysis of the CDPK-SnRK genes in different tissues of *Brassica rapa* and *Arabidopsis thaliana*

**Figure 8:**
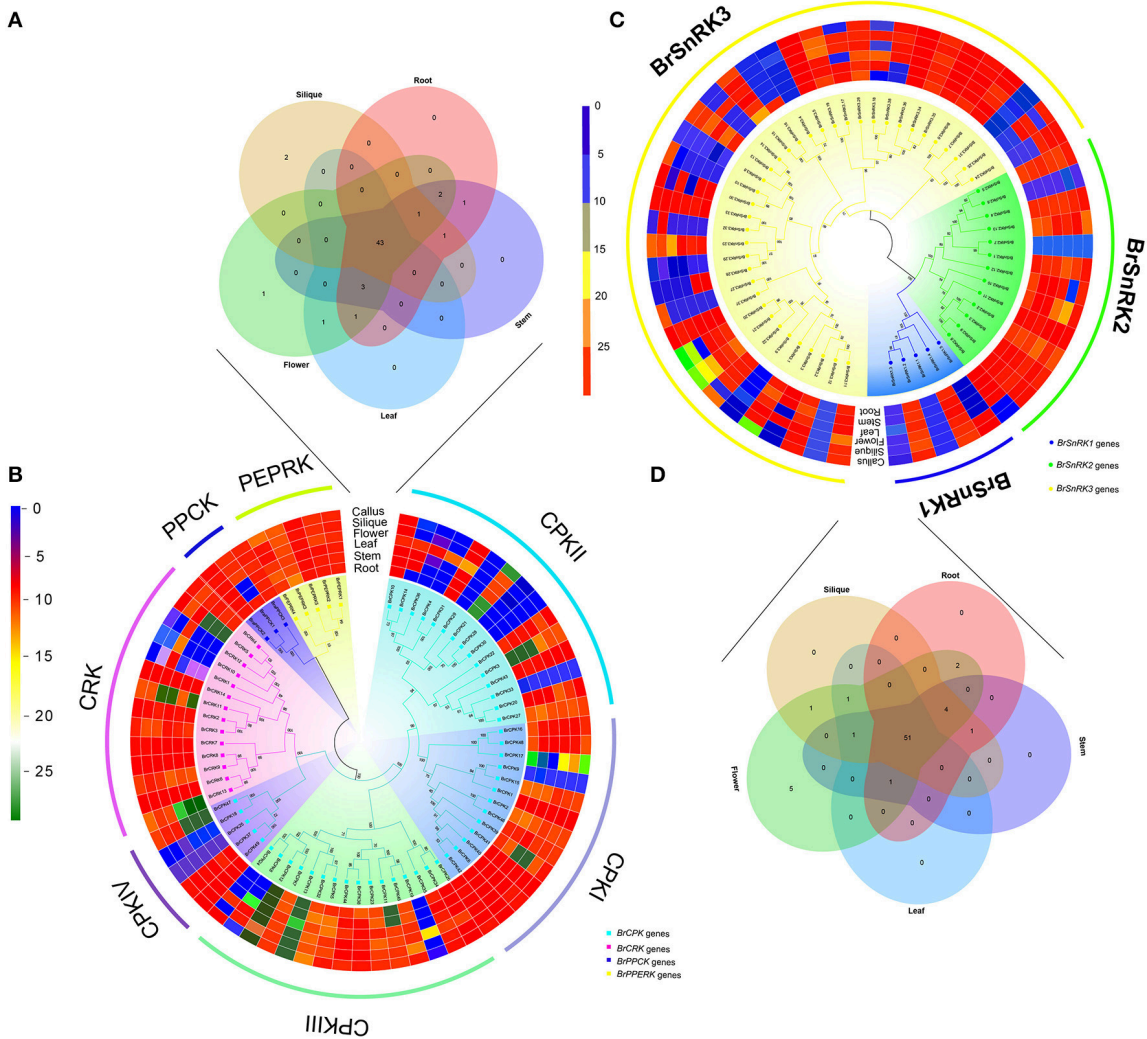
**Analysis of the CDPK-SnRK Genes in different tissues of ***Brassica rapa***. (A,B)** Heat map representation and hierarchical clustering of BrCPK, BrCRK, BrPEPRK, and BrPPCK genes in root, stem, leaf, flower, silique, and callus; Venn diagram depicting the distribution of shared expression of the BrCPK, BrCRK, BrPEPRK, and BrPPCK genes among five *Brassica rapa* tissues. **(C,D)** Heat map representation and hierarchical clustering of BrSnRK genes in root, stem, leaf, flower, silique, and callus; Venn diagram depicting the distribution of shared expression of the BrSnRK genes among five *Brassica rapa* tissues.

To investigate the divergence expression profiles of CDPK-SnRK genes in different organs between *A. thaliana* and *B. rapa*, including roots, stems, leaves, flowers and siliques, the expression patterns of all genes were investigated (Figure S8, Supplementary Tables 5, 6). BrCDPK-SnRK genes were found to be expressed in roots (99 BrCDPK-SnRKs; 77.95%), stems (104; 81.89%), siliques (106; 83.46%), leaves (101; 79.53%), and flowers (119; 93.7%) (Figures 8A–D). A total of 75 (88%) AtCDPK-SnRKs showed high expression (mean-normalized value > 1) in at least one of the five tissues (Figure S8), including roots (34 AtCDPK-SnRKs; 40.00%), stems (36; 42.35%), siliques (22; 25.88%), leaves (19; 22.35%), and flowers (16; 18.23%). Among the 116 CPKs (including 46 AtCPKs and 70 BrCDPKs), 4 (*CPK28, CPK29, CPK30*, and *CPK33*) were not expressed and 2 (*BrCPK4* and *BrCPK25*) were only expressed in flower tissue (Figures 8A,B). In addition, a total of 34 BrCPKs, 8 BrCRKs, 4 BrPEPRKs, and 2 BrPPCKs had high expression levels (FPKM value > 10) in at least one tissue; 13 *BrCPK*s, 2 *BrCRK*s, and 1 *BrPEPRK* were highly expressed in all 5 tissues. However, only 2 SnRK genes were expressed in one tissue; the remains 93 SnRK genes were expressed in five tissues.

Subsequently, we selected the expression patterns of genes in five tissues on the phylogenetic tree of all CDPK-SnRK genes to investigate whether the functions of homologous genes were divergent (Figure S8). All CPKI, PEPRK, and PPCK and most CPKIII and CRK genes had high expression levels, suggesting significant roles of these genes in plant development. Most BrSnRK3s exhibited little or no expression. However, the AtSnRK3s were all expressed in five tissues, indicating that BrSnRK3 genes may have lost some functions after the duplication events (Figure S8).

### Expression divergence and coregulatory networks of CDPK-SnRK genes under multiple treatments in *Brassica rapa*

**Figure 9:**
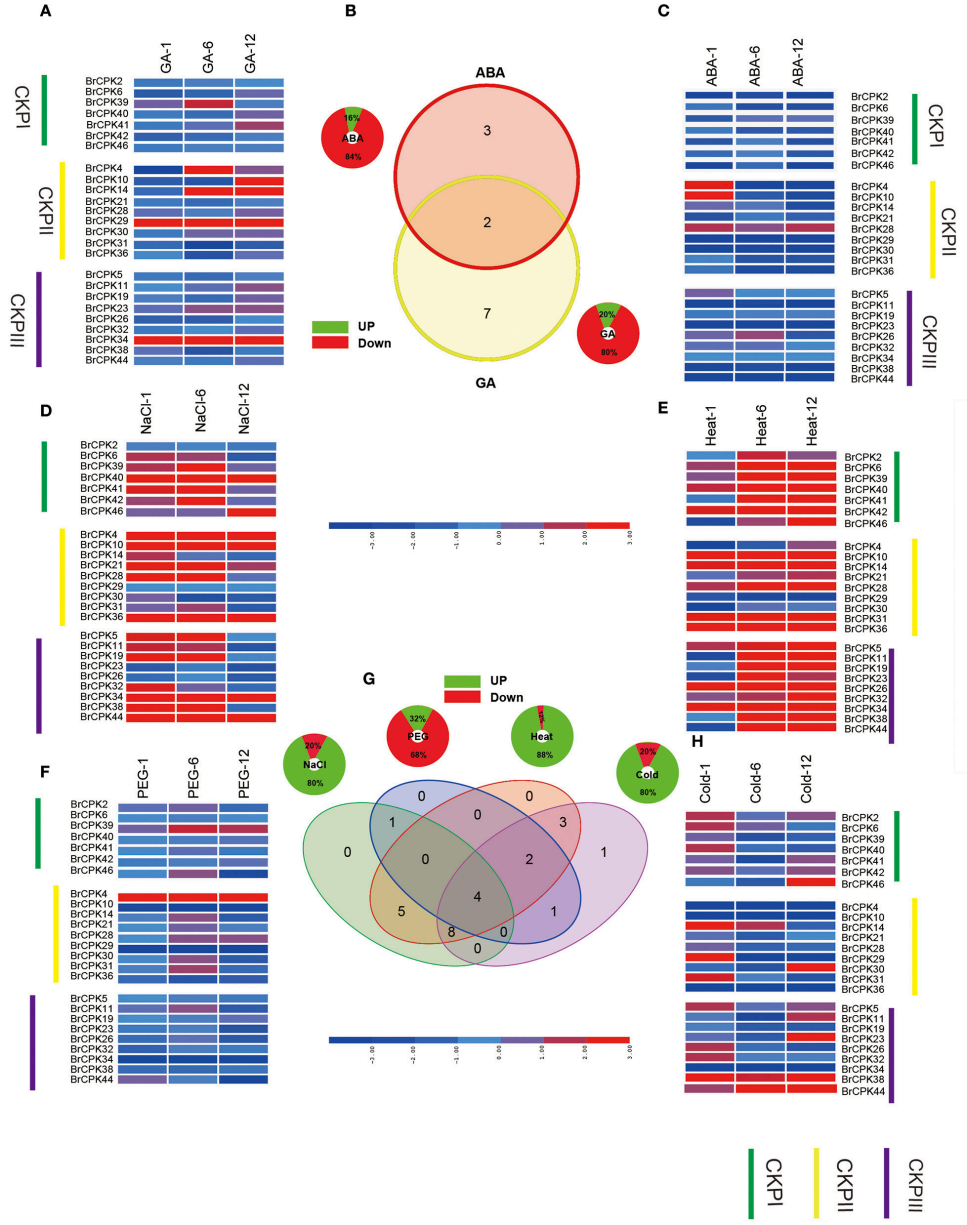
**Expression analysis of the BrCPK genes under six stress treatments in ***Brassica rapa*****. **(A,C–F,H)** Heat map representation the BrCPK genes under six stress treatments, namely, GA, ABA, NaCl, heat, PEG, and cold. **(B,G)** Venn diagram depicting the distribution of shared expression of the BrCPK genes among hormone and abiotic treatments.

**Figure 10:**
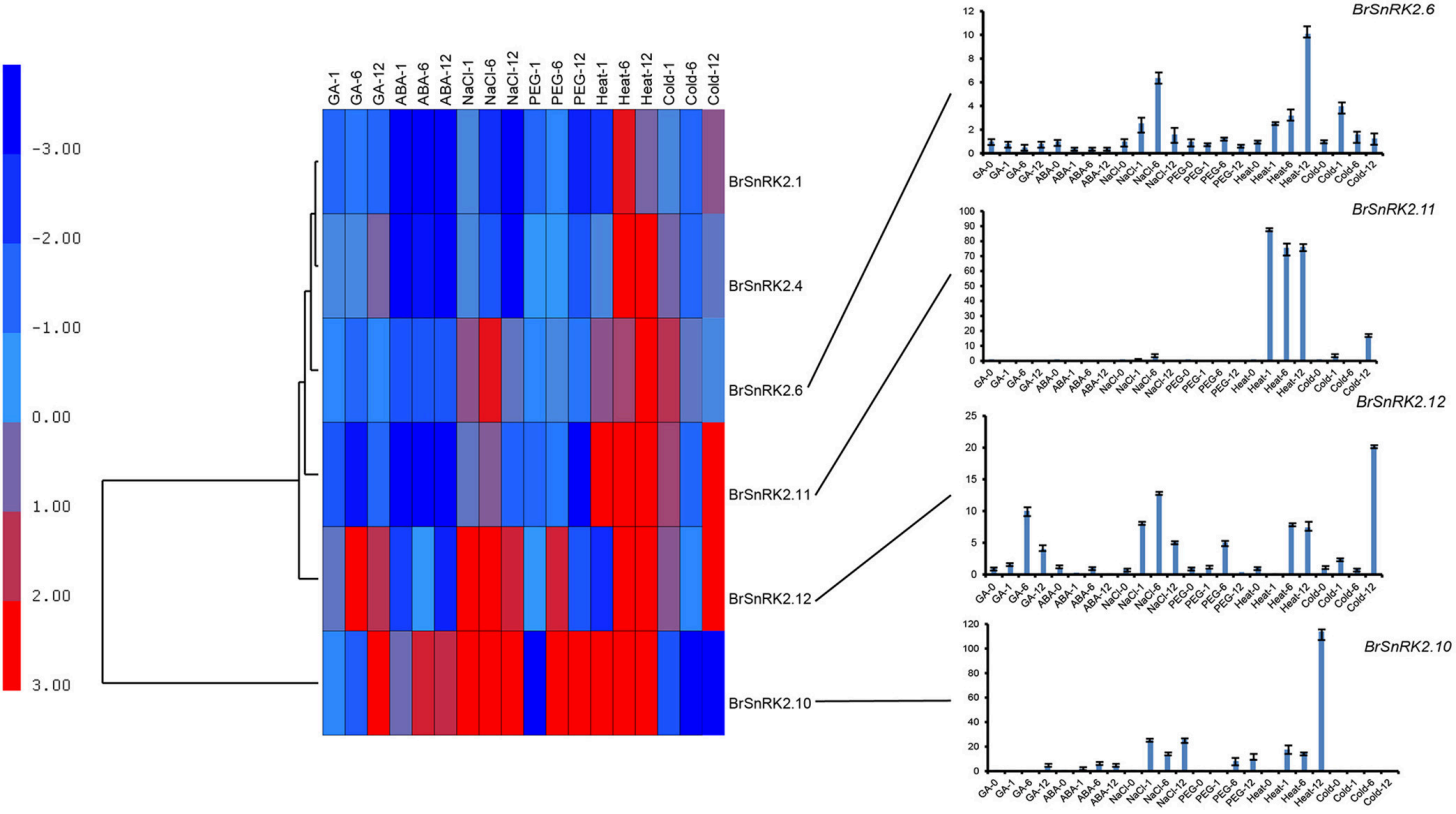
**Expression analysis of the BrSnRK2 genes under six stress treatments in ***Brassica rapa*****. Heat map representation the BrSnRK genes under six stress treatments, namely, GA, ABA, NaCl, heat, PEG, and cold.

CDPK-SnRKs are suggested to play an important role in the regulation of gene expression in response to abiotic stresses (Figure 9, Supplementary Table 7). To investigate the divergence information of the BrCDPK-SnRK gene family, the expression patterns following different treatments, including ABA, GA, NaCl, heat, cold, and PEG treatments, were analyzed (Figures 9, 10). The qRT-PCR results demonstrate that those BrCDPK-SnRK genes respond differentially to particular abiotic stresses. Sixteen percent of investigated BrCPK genes show increased expression levels upon GA at 6 h, while the other 84% of genes display downregulation or no significant changes. Meanwhile, 20% of genes were upregulated at 1 h and 6 h under GA treatment (Figures 9A,C). Two genes (*BrCPK4* and *BrCPK10*) were induced under both ABA and GA treatment (Figure 9B). During four abiotic stress treatments, excluding PEG, 80% of BrCPK genes were highly responsive to cold, NaCl, and heat. We found that, with the exceptions of *BrCPK2, 29, 23* and *26*, all BrCPK genes were significantly induced in response to NaCl treatment (Figures 9D–F,H). Under heat treatment, we found that only two genes (*BrCPK4* and *BrCPK10*) had no expression; other BrCPK genes were highly expressed in NaCl treatment. SnRK2 genes recognized as coding for enzymes involved in abiotic stress signal transduction in plants (Kulik et al., 2011). Therefore, we selected six BrSnRK2 genes; all of these genes had the highest expression under heat treatment (Figure 10). Furthermore, *BrSnRK2.12* was induced under all stress treatments (Figure 10).

**Figure 11:**
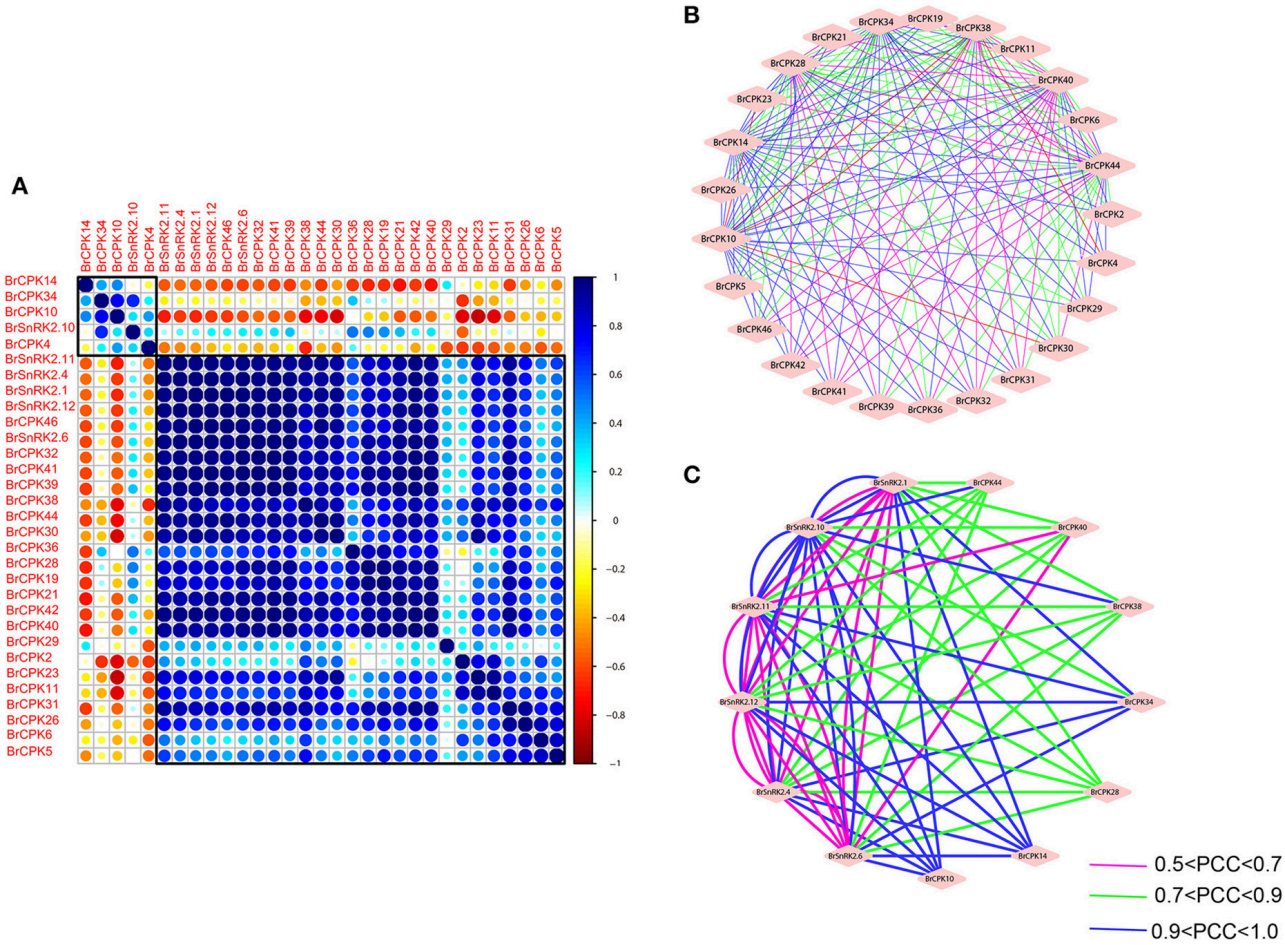
**Correlations and co-regulatory networks of 31 BrCDPK-SnRK genes under stress treatments**. **(A)** Correlation analysis using the R package program. Each correlation is shown by the shades of blue and orange and the size of the circle shape. Blue and orange indicate a positive correlation and negative correlation, respectively. **(B,C)** Co-regulatory networks. The co-regulatory networks of 31 BrCDPK-SnRK genes under stress treatments were established based on the Pearson correlation coefficients (PCCs) of these gene pairs using transformed qPCR data. The PCC of co-regulatory gene pairs was considered significant at the 0.05 significance level (*p*-value), and different line colors and styles indicate the different significance levels of the co-regulated gene pairs.

To investigate the connections between these genes, coregulatory networks were established based on the PCCs of stress-inducible BrCDPK-SnRK gene pairs (Supplementary Table 8, Figure 11). All the BrCDPK-SnRKs appeared to have different degrees of positive correlation. Next, 25 BrCPKs with PCC values that were significant at the 0.05 significance level and were greater than 0.8 were collected and visualized to construct hormone and abiotic stress coregulatory networks (Figure 11A). Six BrSnRKs and seven BrCPKs had positive significant correlations (Figure 11C). Most correlations occurred among members belonging to the same group, suggesting that the gene duplication not only led to functional divergence but also enhanced the cooperative interaction of homologs to help plants to adapt to their complex environment. In addition, based on predicted Chinese cabbage BrCDPK-SnRK superfamily protein interactions, their interactions form a complex network in both *Arabidopsis thaliana* and *B. rapa*. Moreover, to study the protein interactions of stress response genes between *B. rapa* and *Arabidopsis*, STRING 9.1 was utilized (Figure S9). Figure S9 shows four complex interaction networks of CDPK-SnRKs, providing an overall view of the relationship between *B. rapa* and *Arabidopsis*.

## Discussion

In eukaryotes, protein kinases are involved in regulating key aspects of cellular function, including cell division, metabolism, and responses to external signals. CDPK-SnRK plays an important role in stress signal transduction in plants, such as wounding, salt or drought stress (Botella et al., 1996; Patharkar and Cushman, 2000; Saijo et al., 2000), cold (Monroy and Dhindsa, 1995; Saijo et al., 2000), hormone treatment (Abo-El-Saad and Wu, 1995; Botella et al., 1996; Davletova et al., 2001), light (Frattini et al., 1999), and pathogens (Romeis et al., 2001; Murillo et al., 2002).

The gene balance hypothesis predicts that genes whose products participate in signaling networks or macromolecular complexes or are transcription factors are more likely to be retained (Birchler and Veitia, 2007; Aad et al., 2012). In this study, we identified 49 *BrCPKs*, 14 *BrCRKs*, 3 *BrPPCKs*, 5 *BrPEPRKs*, and 56 *BrSnRK*s in the *B. rapa* genome, and they contained a higher rate of copies than the *B. rapa* whole-genome level. This result suggests that these genes had a high degree of retention following WGD. By comparing the number of different duplicated types, gene copies, and distribution of the three subgenomes, we found that all the AtCRK, AtPEPRK, and AtPPCK orthologs were retained in *B. rapa*. In contrast, four AtCPK and two AtSnRK orthologs were completely lost. CRK, PEPRK, and PPCK were more preferentially retained than *CPK* and *SnRK*. CPK (66%) and SnRK (43%) had more than two copies retained in *B. rapa*, and more BrCPK (74%) and BrSnRK (70%) genes experienced segmental duplication. These preferentially retained CDPK-SnRK genes may have more important functions. At the same time, the important functions have been proved in previously researches.

For example, CDPK-SnRKs have been shown to have important roles in various physiological processes, including plant growth and development and abiotic and biotic stress responses in plants. The large number and variety of protein kinases that either have an EF-hand calcium-binding domain in their structure (CPK and CCaMK) or interact with EF-hand proteins (CCaMK, CaMK, CRK, and SnRK3) provides plants with a huge potential for interpreting specific calcium signals and for eliciting specific physiological responses. The SnRK1 kinase plays an important role in carbon-nitrogen interactions (Halford and Paul, 2004; Li et al., 2010) and in the transcription regulation of gene expression. In the developing tuber of potato, the expression level of *SnRK1* was higher, lower in stem and lowest in leaf. The experiment on potato provided evidence for SnRK1 to regulate the transcription (Man et al., 1997). SnRK1 influence starch biosynthesis via regulating the expression of sucrose synthase and the activity of AGPase. SnRK1 activity can respond to sucrose appropriately (Halford and Paul, 2004). Antisense gene expressions in different plant indicate that SnRK1 has very important roles in plant growth and development processes (Halford et al., 2003). The SnRK2s are about 140–160 amino acids shorter than the SnRK1s, averaging about 40 kD in size, and have a characteristic patch of acidic amino acids in their C-terminal domains (Halford et al., 2000). SnRK2 genes, which are a significant part of the ABA signal pathway, are involved in many processes that help plants resistant environmental pressures (Wang et al., 2015). The variety is further amplified in the *SnRK3* subfamily, since these kinases may interact with more than one *CBL/SCaBP* (Guo et al., 2001; Manns et al., 2001; Shi and Eberhart, 2001). Gene expression patterns can provide important clues for gene function. Therefore, the expression patterns of CDPK-SnRK genes under stresses were identified based on qRT-PCR analysis. Overall, expression patterns of CDPK-SnRK genes are dynamic in different tissues during different developmental stages and in response to abiotic stresses, demonstrating their regulatory roles in various cellular processes in *B. rapa*. This finding is consistent with the gene dosage hypothesis.

During evolutionary history, all extant angiosperms genome have undergone at least one and often multiple polyploidization events (Edger and Pires, 2009; Jiao et al., 2011a; Ohno, 2013). *Brassica rapa* experienced a complex WGD history, including γ, α, and β events, and an additional WGT event, providing an excellent chance to study the relationship between gene family fractionation and changes in plant morphotypes (Wang et al., 2011; Cheng et al., 2013). An extensive phylogenetic analysis revealed the evolutionary history of those CDPK-SnRK genes in *B. rapa* and in other plants. In total, 127 CDPK-SnRK genes were grouped in seven classes in *B. rapa*. In addition, we identified CDPK-SnRK genes in 15 other plant species representing the major clades of terrestrial plants. These phylogenetic studies suggest that those identified CPK and SnRK genes were highly conserved in each class across a wide spectrum of plants, indicating their essential regulatory roles in the plant kingdom. For the CPK gene family, the number of genes grouped into each class, gene number and genome size are summarized in Figure 1. Furthermore, there is a difference among the five species in class I, II, III and IV in terms of gene numbers. It suggests that those four classes underwent extensive expansion in angiosperms. Class III was entirely lost during evolution in *V. vinifera*. For the SnRK gene family, two SnRK1 genes were found in *V. carteri*, suggesting that expansion of the SnRK gene family occurred after the divergence of green algae. Meanwhile, SnRK1, SnRK2, and SnRK3 were detected in *Physcomitrella patens*, indicating that the SnRK2 and SnRK3 gene subfamilies appear to be unique to plants, which consist with previously research (Hrabak et al., 2003). Overall, for the six species investigated, the number of CPK and SnRK genes remains different, indicating different evolutionary histories accompanied by rounds of WGD and subsequent gene losses/gains by natural selection constraints.

Most land plants have undergone polyploidization that led to WGD and provided opportunities for duplicated genes to diverge in different evolutionary ways. Each of these genes subsequently experienced one of three fates: subfunctionalization, neofunctionalization, or non-functionalization (deletion or pseudogenization). These fates provided opportunities for duplicated genes to gain functional diversification, resulting in more complex organisms. The *Ka/Ks* substitution rate ratio is an indicator of the selection history on genes or gene regions. Commonly, if the value of *Ka/Ks* is lower than 1, the duplicated gene pairs may have evolved from purifying selection (also called as negative selection); *Ka/Ks* = 1 means neutral selection, while *Ka/Ks*> 1 means positive selection. In this study, the *Ka/Ks* values for the duplicated gene pairs were small (<0.01); thus, it is likely that these pairs have been under purifying selection. Neofunctionalization is an adaptive process during which one copy of a duplicated gene mutates to adopt a novel function that cannot be performed by the ancestral sequence. This mechanism can lead to the retention of both copies over long periods of time. In addition, by comparing the tissue expression pattern among the CDPK-SnRK genes in *B. rapa* and *At. thaliana*, we found that most of the duplicated genes maintained similar expression patterns, but some of the duplicated CDPK-SnRK genes demonstrated higher tissue specialization and diversification, also suggesting that CDPK-SnRKs underwent more neofunctionalization and subfunctionalization. In previous reports, even the most recently diverged paralogs differed in their catalytic efficiency, expression, and/or substrate spectrum, suggesting that duplicates have a relatively high rate of divergence in function.

In this study, we analyzed the evolutionary pattern, gene duplication, and expression divergence of the CDPK-SnRK genes in plants. We conducted a comparative evolutionary analysis with the genome information of selected plants, and our research provides new insight into the evolutionary history of the CDPK-SnRK gene family in plants. For example, CPK had a closer relationship with CRK, indicating that these two may share a common evolutionary origin; and PEPRK appeared between the salicoid duplication and the γ event. By compared expression patterns among tissues, we found that the expansion of CDPK-SnRK genes seems to be associated with increasingly complex organs in the plants' evolution. Under different stress treatments, their coregulatory networks demonstrated that the CDPK-SnRK genes enhanced the cooperative interaction of homologs to adapt to the environment. In conclusion, this study provides useful resources for functional divergence and conservation in the CDPK-SnRK gene superfamily and facilitates our understanding of the effect of polyploidy during the evolution of CDPK-SnRK genes.

## Methods

### Retrieval of sequences

The *B. rapa* sequences were downloaded from BRAD (http://brassicadb.org/brad/) (Wang et al., 2011). The *Arabidopsis* sequences were retrieved from TAIR (http://www.*arabidopsis*.org/), and the sequences of rice were retrieved from RGAP (http://rice.plantbiology.msu.edu/; Kawahara et al., 2013). The gene information on *A. trichopoda* genes was retrieved from the Amborella Genome Database (http://www.amborella.org/). The others 14 species' gene information was downloaded from Phytozome v9.1 (http://www.phytozome.net/; Goodstein et al., 2012). The homologous CDPK-SnRK genes in other species were identified through comparison with *Arabidopsis*. First, BLASTP searches were performed against the rice protein sequences using an *E*-value threshold of 1 × 10^−10^. The top-ranked rice hit was used for BLASTP searches of the *Arabidopsis* proteins to confirm homologies. Starting with both *Arabidopsis* and rice homologs, BLASTP searches were performed against the proteins of other species (*e* < 1 × 10^−10^, identity >70%). These potential sequences were analyzed using the tool SMART (http://smart.embl-heidelberg.de/) and the NCBI database (http://www.ncbi.nlm.nih.gov/).

### Identification of gene synteny and duplicated CDPK-SnRK genes

BLAST and the Multiple Collinearity Scan toolkit (MCScanX) were used for gene synteny analysis according to previous reports (Wang et al., 2012). An all-against-all BLASTP comparison provided the pairwise gene information and the *P*-value for a primary clustering. Paired segments were extended by identifying the clustered genes using dynamic programming. The potentially duplicated genes were also identified using MCScanX. The positions of *B. rapa CDPK-SnRK* genes on the blocks were verified by searching for homologous genes between *A. thaliana* and three subgenomes of *B. rapa* (LF, MF1, and MF2) in BRAD (http://brassicadb.org/brad/searchSynteny.php; Wang et al., 2011). The syntenic diagram was drawn using Circos software (Krzywinski et al., 2009).

### Phylogenetic analysis and characterization of the CDPK-SnRK gene family

Phylogenetic analyses were conducted using MEGA v5.073; maximum-likelihood (ML) trees were constructed with a bootstrap value of 1,000 replications to assess the reliability of the resulting trees. The genetic distance used in this study was also calculated with MEGA v5.0. To identify conserved motifs in the complete amino acid sequences of CDPK-SnRK proteins, we used MEME software (http://meme.sdsc.edu/meme/; Bailey et al., 2009). We researched the gene structure using Gene Structure Display (GSDS, http://gsds.cbi.pku.edu.cn/). The interaction network of CDPK-SnRK proteins in Chinese cabbage was constructed with STRING software (Search Tool for the Retrieval of Interacting Genes/Proteins, http://string-db.org/; Yamada et al., 2011).

### Calculation of the *Ka/Ks* and dating of the duplication events

The duplicated CDPK-SnRK genes from *B. rapa* were aligned using MUSCLE (Edgar, 2004). The protein alignments were translated into coding sequence alignments using an in-house Perl script. *Ks* (synonymous substitution rate) and *Ka* (non-synonymous substitution rate) values were calculated based on the coding sequence alignments using the method of Nei and Gojobori as implemented in KaKs_calculator (Zhang et al., 2006). The *Ks* values of all duplicated *BrCDPK-SnRK* genes were then plotted as the density and a boxplot using the R program (Ihaka and Gentleman, 1996). The divergence time was calculated with the formula T = *Ks/2r*, with *Ks* being the synonymous substitutions per site and r being the rate of divergence for nuclear genes from plants. The value of r was taken to be 1.5 × 10^−8^ synonymous substitutions per site per year for dicotyledonous plants (Koch et al., 2000).

### Expression pattern analysis for *CDPK-SnRK* genes

For expression profiling of the CDPK-SnRK genes in *B. rapa*, we utilized the Illumina RNA-seq data that were previously generated and analyzed by Tong et al. (2013). Six tissues of *B. rapa* accession Chiifu-401-42, including callus, root, stem, leaf, flower, and silique, were analyzed. The transcript abundance was expressed as fragments per kilobase of exon model per million mapped reads (FPKM). The gene expression patterns of each tissue were analyzed using Cluster 3.0, and the expression values were log2 transformed. Finally, heat maps of hierarchical clustering were visualized using Tree View (http://jtreeview.sourceforge.net/). *A. thaliana* development expression profiling was analyzed using the AtGenExpress Visualization Tool (AVT; http://jsp.weigelworld.org/expviz/expviz.jsp) with mean-normalized values (Schmid et al., 2005). Venn diagrams were drawn using the R program.

### Plant material and treatments

Chinese cabbage (Chiifu-401–42) was used for the experiments. The germinated seeds were grown in plastic pots in a 3:1 soil–vermiculite mixture, and the artificial growth conditions were set at 24/16°C, with a photoperiod of 16/8 h for day/night and a relative humidity of 65–70%. Four weeks later, seedlings were subjected to various treatments. For heat and cold treatment, the pots were exposed to 38°C or 4°C; the other growth conditions were same as described earlier. Meanwhile, plants were cultured with the following four treatments: (1) control; (2) 100 μM ABA; (3) 100 μM GA; (4) (w/v) polyethylene glycol (PEG) 6,000, and (5) 250 mM NaCl. All these treatments were performed over a continuous time course (0, 1, 6, 12 h). Each treatment consisted of three biological replicates. All of the samples were frozen in liquid nitrogen and stored at −70°C for RNA preparation.

### RNA isolation and qRT-PCR analyses

Total RNA was isolated from treated frozen leaves using Trizol (Invitrogen, San Diego, CA, USA) according to the manufacturer's instructions. Specific primers used for qRT-PCR were designed by Beacon Designer 7 and are shown in Supplementary Table 9. To check the specificity of the primers, we used BLAST against the *Brassica* genome. The qRT-PCR assays were performed with three biological and technical replicates. The reactions were performed using a StepOnePlus Real-Time PCR System (Applied Biosystems, Carlsbad, CA, USA). qRT-PCR was performed according to a previous report (Song et al., 2013). Relative fold expression changes were calculated using the comparative Ct-value method.

### Pearson correlation analyses

Pearson correlation coefficients (PCCs) of stress-inducible CDPK-SnRK gene pairs were calculated using an in-house Perl script based on log2-transformed quantitative real-time (qRT)-PCR data (Tang et al., 2013). All gene pairs whose PCC was more than 0.8 and was significant at the 0.05 significance level (*P*-value) were collected for a gene coregulatory network analysis. The coexpression networks were graphically visualized using Cytoscape based on the PCCs of these gene pairs (Shannon et al., 2003).

## Author contributions

PW was responsible for the experimental design, data analysis, and manuscript writing. WD, WW, YL contributed to the experimental work. XH contributed to the interpretation of results and coordinated the study. All authors read and approved the final manuscript.

### Conflict of interest statement

The authors declare that the research was conducted in the absence of any commercial or financial relationships that could be construed as a potential conflict of interest.
