# Supplementary material for: Comprehensive Analysis of the CDPK-SnRK Superfamily Genes in Chinese Cabbage and Its Evolutionary Implications in Plants
Source: Front Plant Sci. 2017 Feb 10;8:162. doi: 10.3389/fpls.2017.00162 (PMC5301275; doi:10.3389/fpls.2017.00162)
Supplement: Supplementary file 1 [file Presentation1.pdf]

Comprehensive Analysis of the CDPK-SnRK Superfamily Genes in Chinese Cabbage and Its Evolutionary Implications in Plants

Peng Wu[1] , Wenli Wang[1] , Weike Duan[1,2] , Ying Li[1] , Xilin Hou[1*]

[1]State Key Laboratory of Crop Genetics and Germplasm Enhancement/Key Laboratory of Biology and Germplasm Enhancement of Horticultural Crops in East China, Ministry of Agriculture, Nanjing Agricultural University, Nanjing 210095, China

[2] School of Life Science and Food Engineering, Huaiyin Institute of Technology, Huaian, China

*To whom correspondence should be addressed: hxl@njau.edu.cn

Tel.: +86 025 8439 5917

Figure S1 Schematic diagram of amino acid motifs and gene structures of *BrCDPK-SnRK* genes in *Arabidopsis* and *B. rapa*.

Figure S2. **Alignments of *A. thaliana* and *B.rapa CPK, CRK, PEPRK, PPCK* and SnRK proteins.** Conservations and consensus were demonstrated under the alignments.

Figure S3. The phylogenetic trees of the *BrCDPK-SnRK* genes from 13 Angiospermae.

Figure S4 (**a**) The phylogenetic tree of the *BrCDPK-SnRK* genes from 13 Angiospermae, one Pteridophyta, one Bryophyta and two Chlorophyta species. (**b**) The genetic distance among clades *CPK, CRK, PEPRK, PPCK* and *SnRK*.

Figure S5 Information of *CPK, CRK, PEPRK, PPCK* gene family clades in *Brassica rapa, Arabidopsis thaliana, Carica papaya, Populus trichocarpa, Vitis vinifera* and *Amborella trichopoda*. The α, β, γ, and salicoid duplications and the Brassica-specific triplication are indicated on the branches of the trees according to the Plant Genome Duplication Database. To classify these *CPK, CRK, PEPRK, PPCK* genes, phylogenetic trees with *CPK, CRK, PEPRK,*

PPCK genes for each of the five species by maximum likelihood using MEGA5, respectively.

Figure S6 Information of *SnRK* gene family clades in *Brassica rapa*, *Arabidopsis thaliana*, *Carica papaya*, *Populus trichocarpa*, *Vitis vinifera* and *Amborella trichopoda*. The α, β, γ, and salicoid duplications and the Brassica-specific triplication are indicated on the branches of the trees according to the Plant Genome Duplication Database. To classify these *SnRK* genes, phylogenetic trees with *SnRK* genes for each of the five species by maximum likelihood using MEGA5, respectively.

Figure S7. **Phylogenetic analysis of the *CPK* gene family in angiosperms.** Protein sequences were aligned by ClustalW2. A phylogenetic tree was constructed by maximum likelihood (ML) and bootstrap values were calculated with 1000 replications using MEGA5.2. Gene losses are possibly indicated by X. α, β, and γ duplications as well as the Brassica triplication are indicated. green pentagons indicate the Brassica triplication.

Fig. S8. Expression patterns analysis of the *CDPK-SnRK* genes in *Arabidopsis thaliana* and *Brassica rapa*. Expression patterns of the *CPK, CRK, PEPRK*, *PPCK* and *SnRK* in five tissues, including the root, steam, leaf, flower and silique. Gene expression was determined using the AtGenExpress Visualization Tool and *B. rapa* RNA-Seq data. The bar at the bottom of each heat map represents the relative expression values.

Fig. S9. Interaction network of *CDPK-SnRK* genes in betweet *Arabidopsis thaliana and B.rapa.* (**a,b,c,d**) Specific protein interactions of *CPK, SnRK* in *A. thaliana and B.rapa* were constructed using STRING (Search Tool for the Retrieval of Interacting Genes/Proteins; http://string-db.org/).
