# Supplementary figures and images for: Comprehensive Analysis of the CDPK-SnRK Superfamily Genes in Chinese Cabbage and Its Evolutionary Implications in Plants
Source: Front Plant Sci. 2017 Feb 10;8:162. doi: 10.3389/fpls.2017.00162 (PMC5301275; doi:10.3389/fpls.2017.00162)

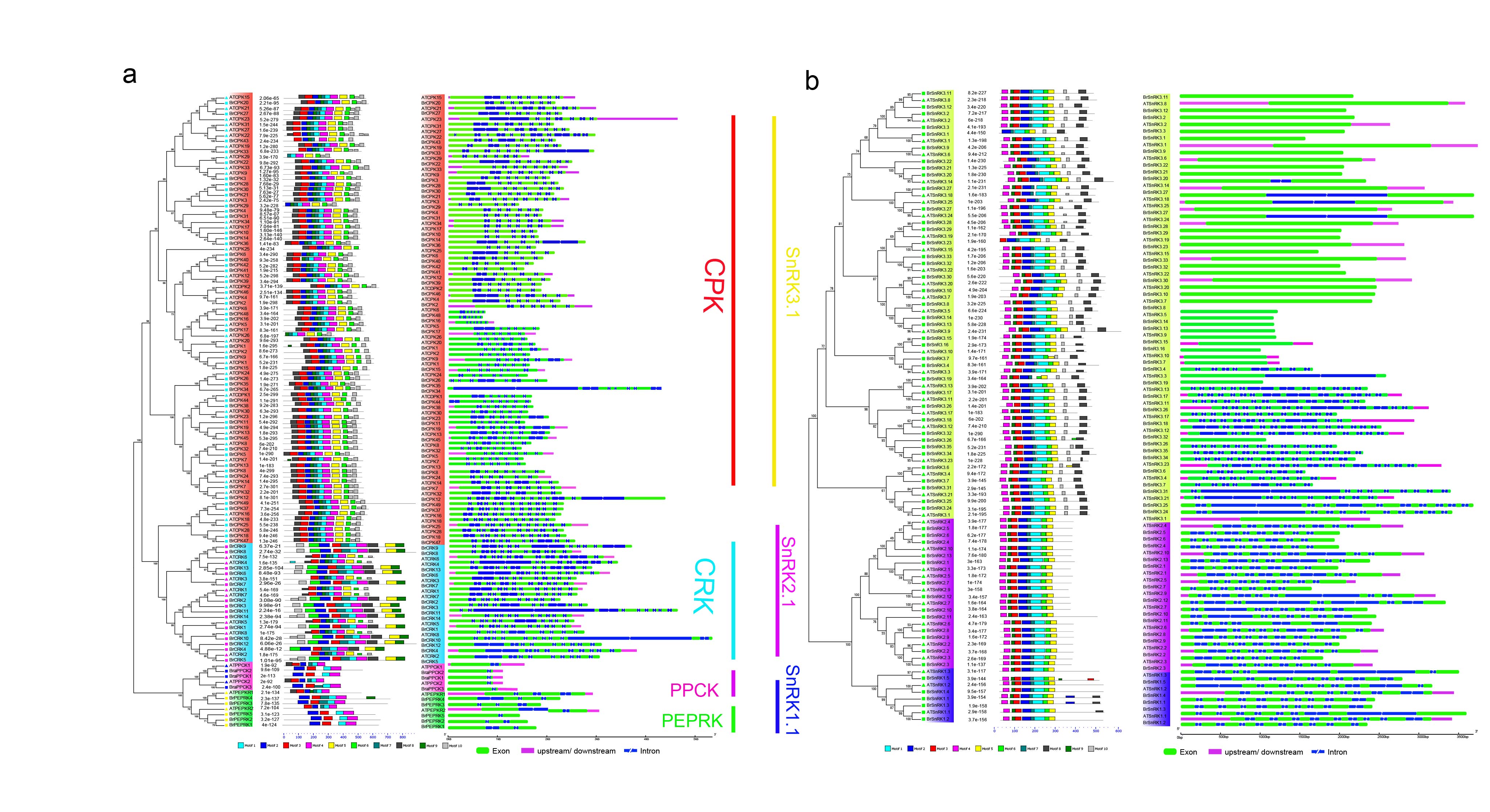

Supplement: Supplementary file 3 [file Image1.JPEG]

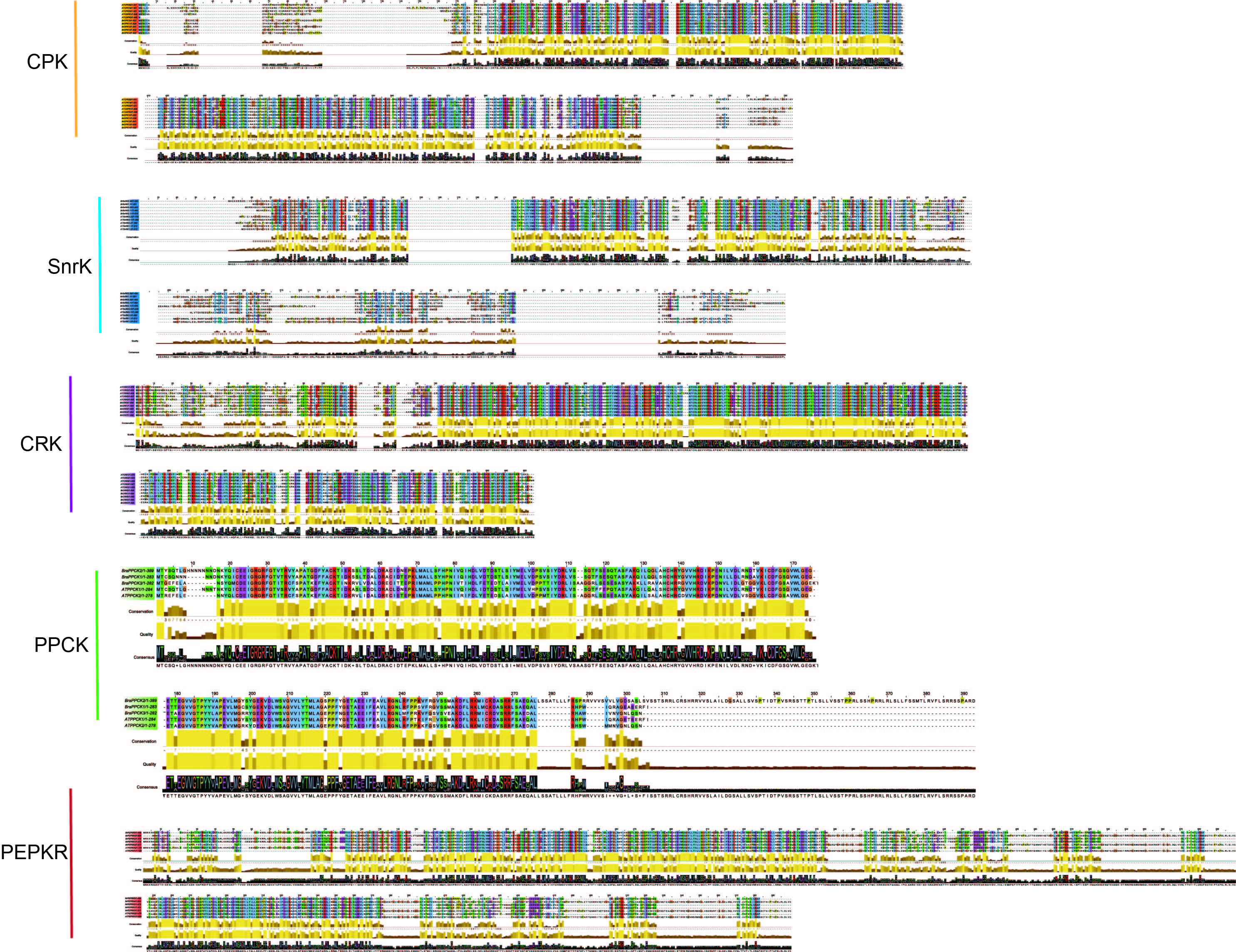

Supplement: Supplementary file 4 [file Image2.JPEG]

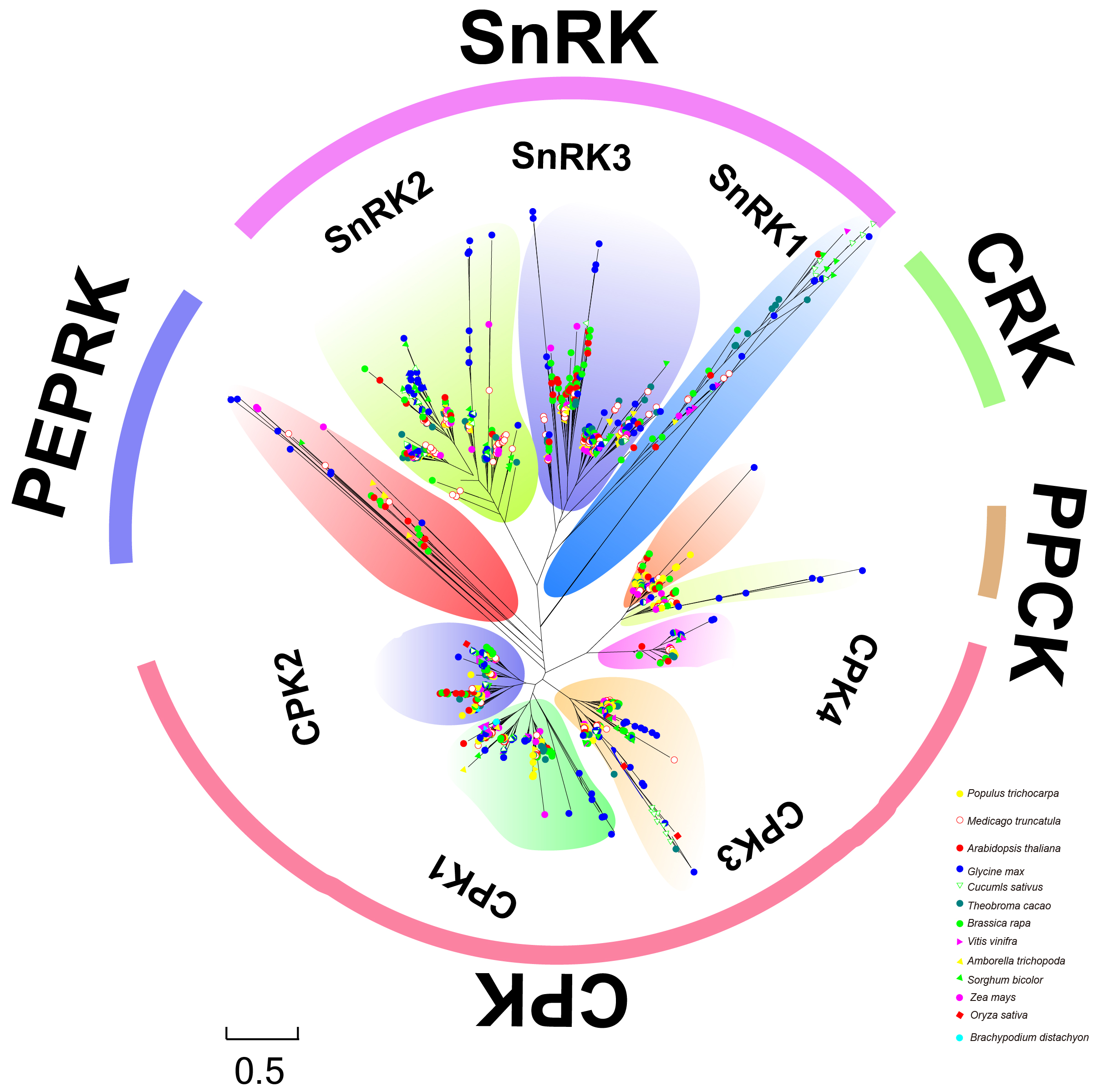

Supplement: Supplementary file 5 [file Image3.JPEG]

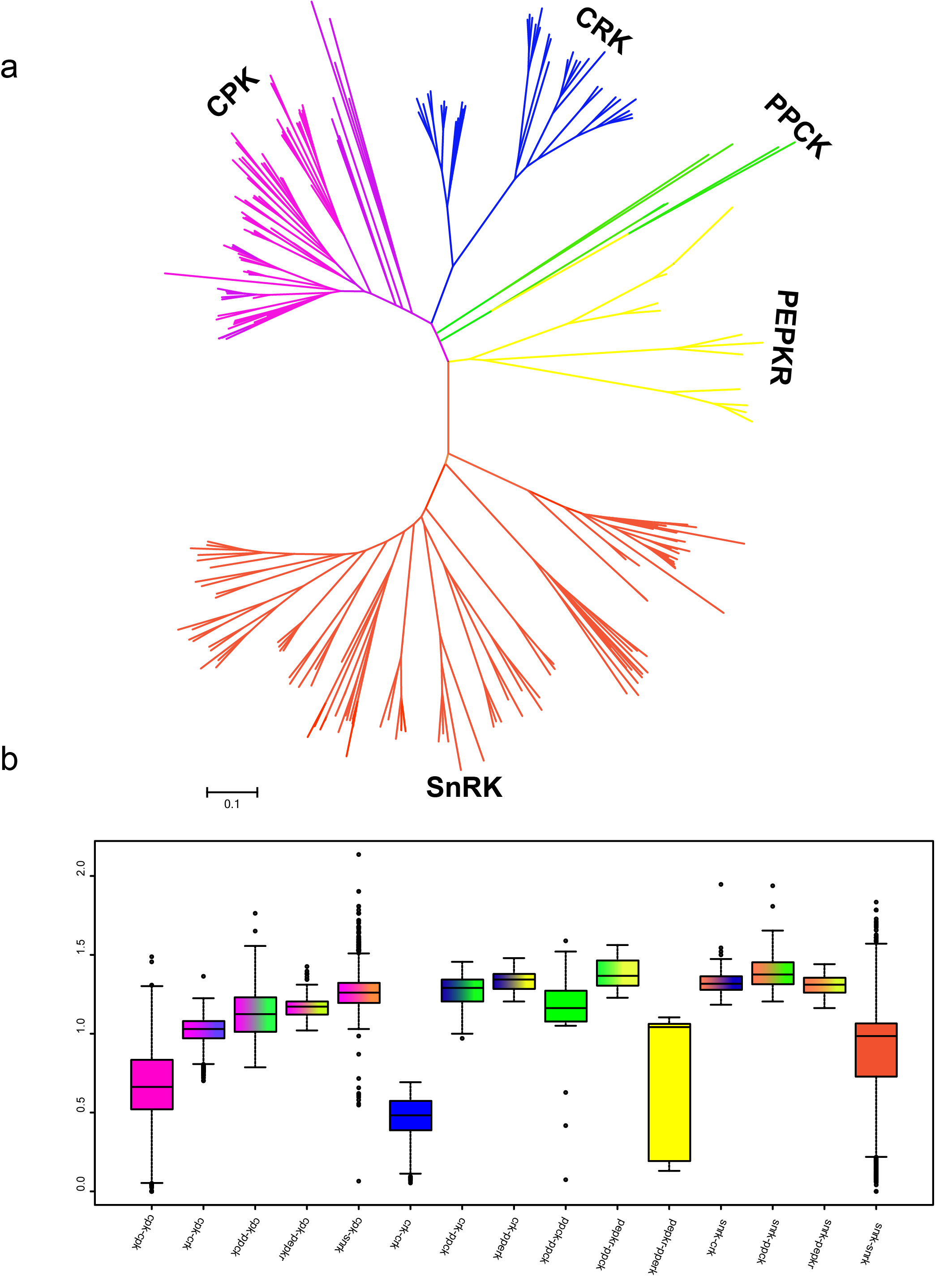

Supplement: Supplementary file 6 [file Image4.JPEG]

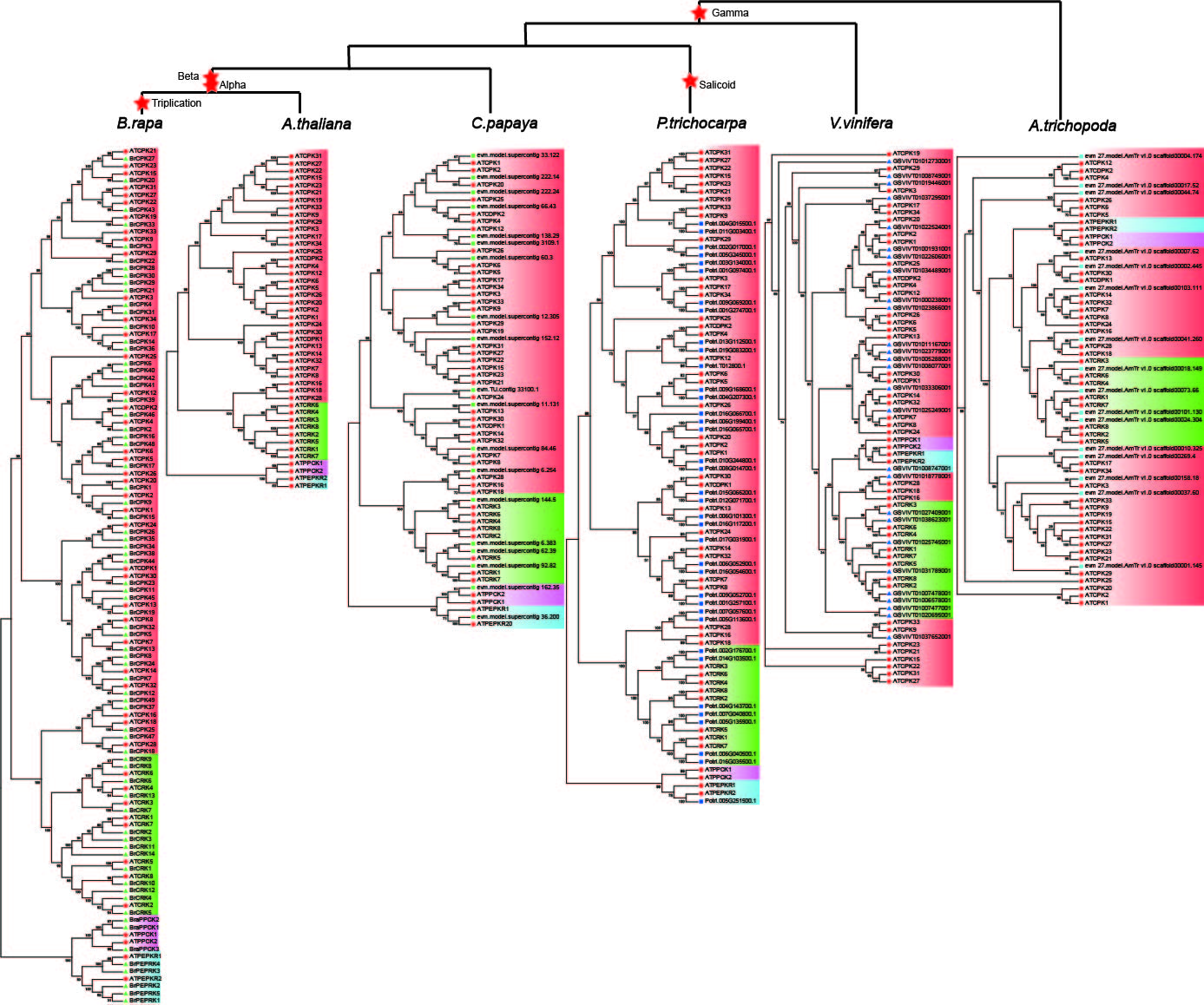

Supplement: Supplementary file 7 [file Image5.JPEG]

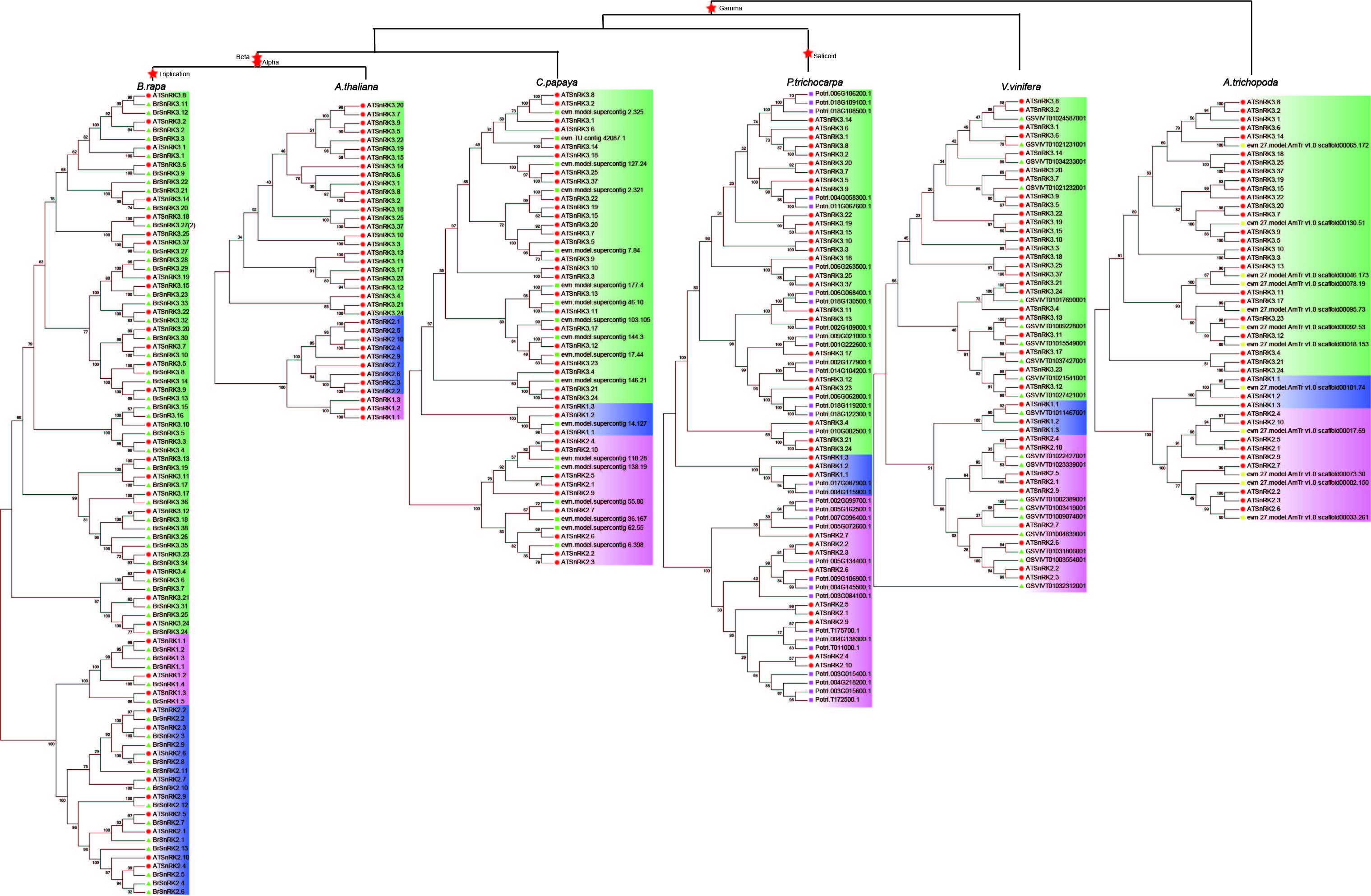

Supplement: Supplementary file 8 [file Image6.JPEG]

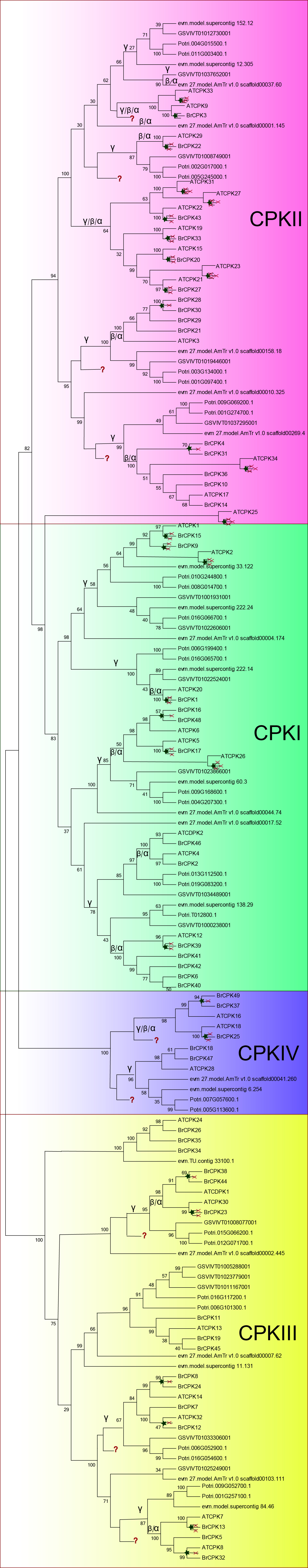

Supplement: Supplementary file 9 [file Image7.JPEG]

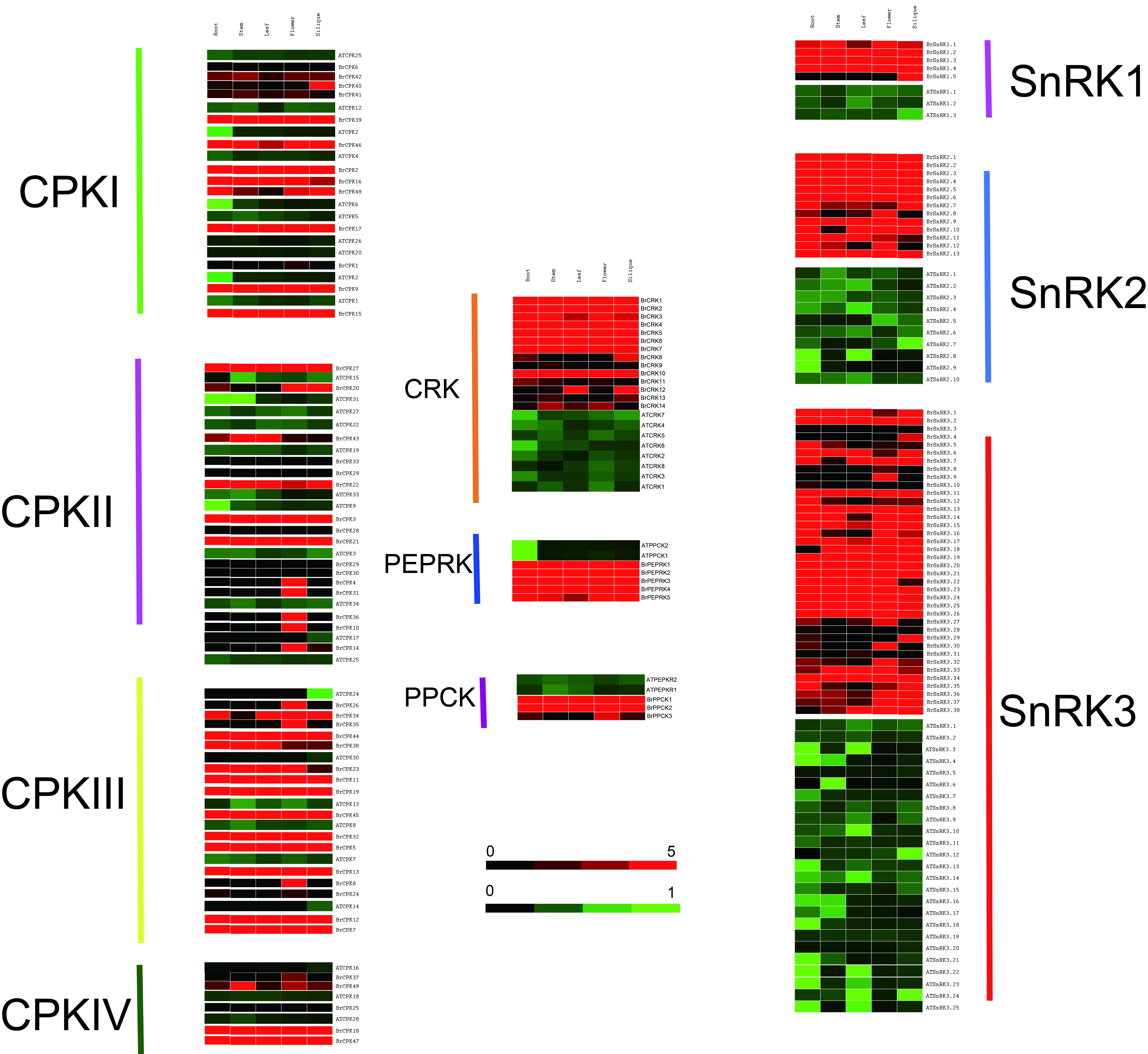

Supplement: Supplementary file 10 [file Image8.JPEG]

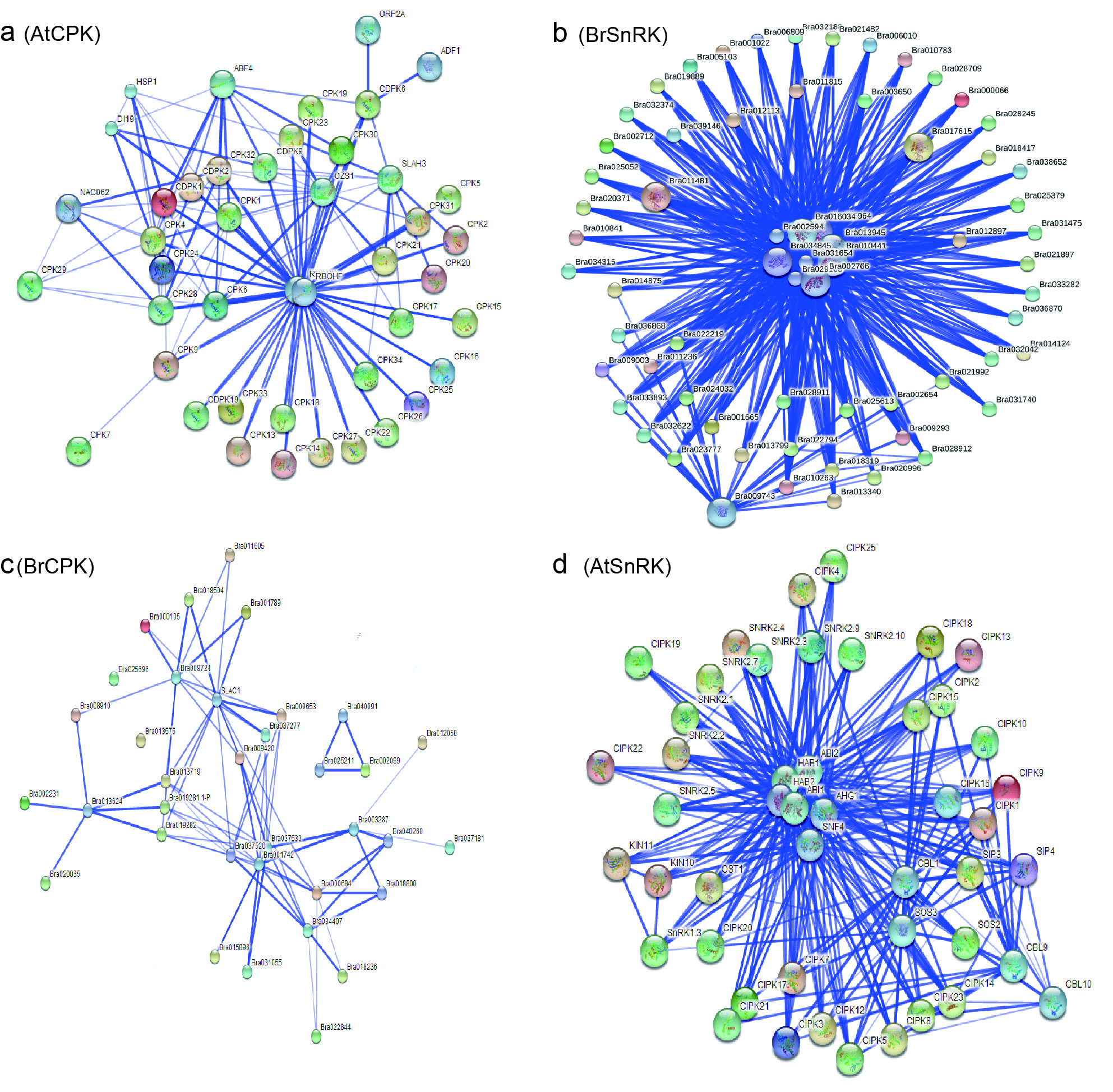

Supplement: Supplementary file 11 [file Image9.JPEG]
